# Impact of Everolimus Initiation and Corticosteroid Weaning During Acute Phase After Heart Transplantation on Clinical Outcome: Data from the Korean Organ Transplant Registry (KOTRY)

**DOI:** 10.3389/ti.2024.11878

**Published:** 2024-04-05

**Authors:** Kyu-Sun Lee, Hyungseop Kim, Sun Hwa Lee, Dong-Ju Choi, Minjae Yoon, Eun-Seok Jeon, Jin-Oh Choi, Jeehoon Kang, Hae-Young Lee, Sung-Ho Jung, Jaewon Oh, Seok-Min Kang, Soo Yong Lee, Min Ho Ju, Jae-Joong Kim, Myoung Soo Kim, Hyun-Jai Cho

**Affiliations:** ^1^ Department of Internal Medicine and Division of Cardiology, Eulji University Hospital and Eulji University School of Medicine, Daejeon, Republic of Korea; ^2^ Department of Internal Medicine, Seoul National University Hospital and Seoul National University College of Medicine, Seoul, Republic of Korea; ^3^ Division of Cardiology, Keimyung University Dongsan Medical Center, Daegu, Republic of Korea; ^4^ Cardiovascular Center, Department of Internal Medicine, Seoul National University Bundang Hospital, Seoul National University College of Medicine, Seongnam, Republic of Korea; ^5^ Department of Internal Medicine, Samsung Medical Center, Sungkyunkwan University College of Medicine, Seoul, Republic of Korea; ^6^ Department of Thoracic and Cardiovascular Surgery, Asan Medical Center, University of Ulsan College of Medicine, Seoul, Republic of Korea; ^7^ Department of Internal Medicine, Yonsei University College of Medicine, Seoul, Republic of Korea; ^8^ Division of Cardiology, Department of Internal Medicine, Pusan National University Yangsan Hospital, Yangsan, Republic of Korea; ^9^ Department of Thoracic and Cardiovascular Surgery, Pusan National University Yangsan Hospital, Medical Research Institute, Pusan National University School of Medicine, Yangsan, Republic of Korea; ^10^ Department of Internal Medicine, Asan Medical Center, University of Ulsan College of Medicine Seoul, Seoul, Republic of Korea; ^11^ Deparment of Surgery, Yonsei University College of Medicine, Seoul, Republic of Korea

**Keywords:** heart transplantation, mTOR inhibitor, Korean Organ Transplant Registry, steroid weaning, primary outcome, rejection, cardiac allograft vasculopathy

## Abstract

The effect of changes in immunosuppressive therapy during the acute phase post-heart transplantation (HTx) on clinical outcomes remains unclear. This study aimed to investigate the effects of changes in immunosuppressive therapy by corticosteroid (CS) weaning and everolimus (EVR) initiation during the first year post-HTx on clinical outcomes. We analyzed 622 recipients registered in the Korean Organ Transplant Registry (KOTRY) between January 2014 and December 2021. The median age at HTx was 56 years (interquartile range [IQR], 45–62), and the median follow-up time was 3.9 years (IQR 2.0–5.1). The early EVR initiation within the first year post-HTx and maintenance during the follow-up is associated with reduced the risk of primary composite outcome (all-cause mortality or re-transplantation) (HR, 0.24; 95% CI 0.09–0.68; *p* < 0.001) and cardiac allograft vasculopathy (CAV) (HR, 0.39; 95% CI 0.19–0.79; *p* = 0.009) compared with EVR-free or EVR intermittent treatment regimen, regardless of CS weaning. However, the early EVR initiation tends to increase the risk of acute allograft rejection compared with EVR-free or EVR intermittent treatment.

## Graphical Abstract

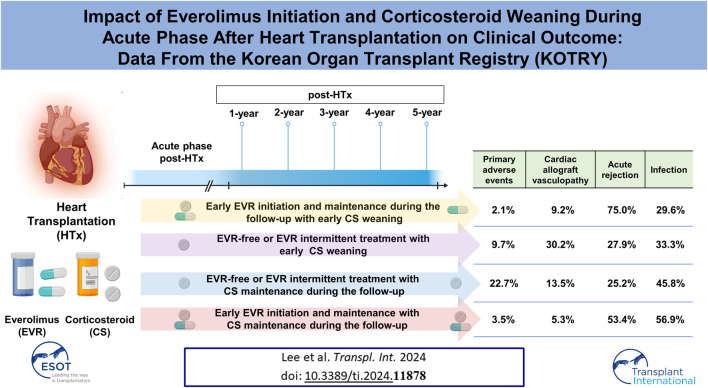

## Introduction

Calcineurin inhibitors (CNI), mycophenolic acid (MPA), mammalian target of rapamycin (mTOR) inhibitors, and corticosteroids (CS) are the main choices for immunosuppressive therapy after heart transplantation (HTx) [1, 2]. Advanced maintenance regimens consisting of immunosuppressive agents and therapeutic drug monitoring post-HTx contribute to the increased success of HTx by reducing the risk of rejection [3, 4]. However, temporal changes of regimens or dosages in immunosuppressive agents are still associated with a risk of acute rejection after transplantation, while inappropriate administration leads to adverse drug effects [5–7]. Therefore, the principal goal of immunosuppressive therapy is to balance the prevention of allograft rejection and adverse immunotherapeutic effects [8]. In this context, determining the optimal timing of the treatment initiation or change in immunosuppressant dosage is crucial to maximize efficacy and minimize adverse effects.

A previous study has reported the safety of tacrolimus (TAC) monotherapy compared with TAC and mycophenolate mofetil (MMF) therapy and CS withdrawal in the early phase post-transplantation [9]. Subsequently, recent studies have reported the safety and efficacy of mTOR inhibitor treatment initiation with CNI tapering or withdrawal in HTx recipients [10–15]. However, these studies have evaluated the efficacy and safety of single immunosuppressive agents. Initiation, adjustment, and changes in immunosuppressive agents are inevitable, depending on various factors, including drug adverse effects or tolerability during the acute phase post-transplantation. Against this background, the impact of concurrent changes in immunosuppressive agents with initiation, tapering, or withdrawal during the acute phase post-HTx on clinical outcomes remains to be determined. Therefore, this study aimed to evaluate whether temporal changes in the immunosuppressive agents during the acute phase post-HTx are associated with clinical outcomes in HTx recipients by using heart transplant cohort database of the Korean Organ Transplant Registry (KOTRY).

## Patients and Methods

### Data Source and Collection

The KOTRY is the first nationwide prospective cohort study of solid organ transplantation launched in 2014 [16]. The KOTRY consist of five organ-transplant cohorts (kidney, liver, lung, pancreas, and heart). Among cohorts, 7 hospitals (Seoul National University Hospital, Samsung Medical Center, Asan Medical Center, Seoul National University Bundang Hospital, Yonsei Severance Hospital, Keimyung University Dongsan Medical Center, Pusan National University Yangsan Hospital) are participating in the heart transplant cohort. After written informed consent was obtained from each recipient prior to HTx, HTx recipients from representative medical centers have been consecutively enrolled in the KOTRY^1^ upon transplantation, and recipient-related data for study have been prospectively recorded. Detailed information regarding the collected data and the definition of comorbidities are described in the first and second reports of the Korean Heart Transplant Registry [17, 18]. Briefly, recipients enrolled in heart transplant cohort of the KOTRY are followed up at 1, 6, 12, 24, 36, 48, 60, and 120 months to monitor for rejection and screen for adverse events post-HTx according to the heart transplant cohort protocol. We annually collected the data including [1] the recipient’s vital signs and comorbidities [2]; the information about prescribed medications and changes to medications including immunosuppressants [3]; a laboratory test [4]; PRA (panel reactive antibody) I & II [5]; DSA (donor specific antibodies) [6]; echocardiographic assessment [7]; recent events (death, rejection, cardiac allograft vasculopathy, renal replacement therapy or re-transplantation); and [8] post-transplantation complications (rejection, malignancy, diabetes mellitus, hypertension, stroke, infection, skeletal complication, and renal impairment).

Endomyocardial biopsy (EMB) was performed according to center-specific protocol biopsy for rejection surveillance. Typically, KOTRY protocol recommended EMBs within 30 days, 3, 6, 12, and 24 months after HTx. However, the specific timing and frequency of EMBs could vary slightly based on the center established protocol. After 24 months, routine EMBs were generally discontinued. Additional EMBs could be performed beyond 2 years if clinically indicated. The decision to perform an EMB was based on individual patient factors and clinical evaluation such as any suspicion of symptomatic allograft rejection, even if other tests are inconclusive. After all biopsies performed during the follow-up period were reviewed, only those cases where rejection was confirmed were recorded in the cohort. Further, if EMB was performed at a similar time as the protocol biopsy, the reason was clearly recorded at electronic case report form (eCRF).

Cardiac allograft vasculopathy (CAV) defined as abnormal coronary angiography findings diagnosed either by coronary angiography (CAG) with or without IVUS (adjunct intravascular imaging can be considered if expertise is available), or CT coronary angiography and graded using the international society of heart and lung transplantation (ISHLT) nomenclature [19, 20]. KOTRY heart transplant protocol recommended routine CAG at 12 months post-transplant. If CAG detected any abnormality, IVUS was further recommended for detailed assessment. Beyond 12 months, coronary evaluation was recommended annually through either CAG or CT-CAG. Additionally, coronary evaluation was performed (regardless of the one-year schedule) if there is clinical suspicion of cardiac allograft vasculopathy (CAV) during the follow-up period. This result was selected and recorded at eCRF during the follow-up period.

### Data Quality

Data management of heart transplant cohort of the KOTRY was performed by using a web-based electronic case report form (eCRF) with Pharmacoepidemiology and Clinical Trial Applications X (PhactaX) system, which was developed by the Medical Research Collaborating Center of Seoul National University Hospital. All participating sites received multiple onsite monitoring visits to verify informed consent in all participants and to check key data in enrolled patients. All clinical data were collected and recorded in eCRF (version 2.7) compliant centralized online database. In addition to checking for outliers via automated computational methods, data quality was verified every 3 months by verifying values entered in the database against the primary source documents.

### Study Population

From 2014 to 2021, 813 HTx recipients aged above 18 years were enrolled in the KOTRY HTx database. To evaluate the effect of the early EVR initiation and CS weaning within the first year post-HTx on clinical outcome, we excluded the following recipients from this study. 64 recipients were excluded due to insufficient follow-up (less than 1 month post-HTx). 56 recipients who died within the first year post-HTx were excluded. 73 recipients were excluded due to missing data on the presence of EVR and CS prescription, dose, or trough levels during the follow-up. Finally, 620 HTx recipients were included in this study. Written informed consent was obtained from each participant. The use of the registry data for this study was approved by the institutional review board of Seoul National University Hospital (IRB No. 1406-082-588).

### Study Outcomes

The primary outcome was a composite of all-cause death or re-transplantation. Secondary outcomes were cardiac allograft vasculopathy (CAV), acute allograft rejection, infection, and malignancy during the follow-up period. The diagnosis of acute allograft rejection was based on endomyocardial biopsy (EMB) findings by an experienced pathologist following ISHLT guidelines [21, 22]. The acute allograft rejection was classified into acute cellular rejection (ACR) (Grade 1R, Grade 2R, Grade 3R, and Unspecified) and acute antibody-mediated rejection (AMR). The pathologic grading and reporting of AMR were as follows: pAMR 1 (pAMR 1-histopathologic, pAMR 1-immunopathologic), pAMR 2, pAMR 3, unspecified.

CAV was classified as insignificant (CAV 0), mild (CAV 1), moderate (CAV 2), or severe (CAV 3) according to the International Society for Heart and Lung Transplantation (ISHLT) CAV grading report [19]. The infection was defined as the cases requiring hospitalization due to pathogens such as viruses, bacteria, fungi, or parasites and was diagnosed by signs or symptoms related to infection and detection of pathogen by laboratory tests. Malignancies included the following diseases diagnosed during the follow-up period: malignancy of skin, renal, urogenital, respiratory, upper/lower gastrointestinal, hepatobiliary-pancreas, gynecologic, breast, hematologic, intracranial, and thyroid.

### Statistical Analysis

Descriptive statistics were used to describe and summarize patients’ baseline characteristics and comorbidities. Categorical variables were compared using the chi-square or Fisher’s exact test. Continuous variables are presented as mean ± standard deviation or medians (25th−75th percentiles), and group differences were compared using Student’s *t* or the Mann-Whitney test.

The Cox proportional hazard regression model was used to calculate hazard ratios (HR) and 95% confidence intervals (CI). For the comparison of clinical outcomes regarding changes in immunosuppressive agents or regimens, a multivariate Cox proportional hazard regression model was used to calculate adjusted HR and its CI. The following variables were included for adjustment: age, sex, diabetes mellitus, hypertension, smoking, donor-specific antibody, and desensitization. The cumulative incidence of primary and secondary outcomes was estimated using the Kaplan–Meier method, and the log-rank test was used to evaluate differences between groups. Statistical significance was set at *p* < 0.05. SPSS Statistics version 25.0 (IBM, Chicago, IL, United States) was used for statistical analyses.

## Results

### Recipient and Donor Characteristics

The baseline characteristics of recipients and donors are presented in Table 1. The median recipient age was 56 years, and 69.2% were male. Of the recipients, 169 (27.3%) had diabetes mellitus, 196 (31.6%) had hypertension, and 100 (16.0%) had chronic kidney disease. The mean left ventricular ejection fraction (LVEF) at HTx was 27.1%. Cardiomyopathy (59.4%) was the most frequent cause of HTx, followed by ischemic heart disease (19.8%). In total, 516 (83.2%) patients required inotropic support to stabilize circulation. A total of 162 patients (26.1%) received venoarterial extracorporeal membrane oxygenation (VA-ECMO), and 46 (7.4%) received ventricular assist device therapy as a bridge to HTx. A total of 129 patients (20.8%) received mechanical ventilation, and 106 (17.1%) received mechanical ventilation in combination with VA-ECMO. In total, 124 (20.1%) patients exhibited class I and/or class II pre-transplantation panel reactive antibodies against human leukocyte antigen (anti-HLA) greater than 50%. 74 patients (13.4%) had donor-specific anti-HLA antibodies, and 50 (8.1%) were desensitized before transplantation. The median operative time was 339 min, and the median warm ischemic time was 51 min. A total of 54 patients (8.7%) received VA-ECMO, and 103 (16.6%) received continuous renal replacement therapy after transplantation.

**TABLE 1:** Baseline characteristics of HTx recipients and donors.

Variables	Overall (*N* = 622)
Recipient characteristics	
Age (years), median (IQR)	56.0 (45.0–62.0)
Sex (male), no. (%)	429 (69.2)
BMI (kg/m^2^)	22.6 ± 3.5
Diabetes mellitus, no. (%)	169 (27.3)
Type 1 DM	4 (0.6)
Type 2 DM without insulin	138 (22.3)
Type 2 DM with insulin	27 (4.4)
Hypertension, no. (%)	196 (31.6)
Smoking status, no. (%)	
Never	360 (58.1)
Current	59 (9.5)
Former	197 (31.8)
Previous malignancy, no. (%)	49 (7.9)
Chronic kidney disease, no. (%)	100 (16.1)
CKD stage 3 (eGFR 30–59)	66 (10.6)
CKD stage 4 (eGFR 15–29)	8 (1.3)
CKD stage 5 (eGFR <15) with HD	26 (4.2)
Left ventricular ejection fraction (%)	27.1 ± 14.2
Lab findings at heart transplantation	
WBC (×10^3^ μL/L)	6.8 (5.3–9.0)
Hemoglobin (g/dL)	11.0 (9.5–12.8)
Platelet (×10^3^/μL)	161.0 (112.0–218.0)
BUN (mg/dL)	20.3 (15.1–29.3)
Creatinine (mg/dL)	1.0 (0.8–1.4)
Total cholesterol (mg/dL)	134.0 (108.0–163.0)
LDL-C (mg/dL)	76.5 (57.0–104.0)
HDL-C (mg/dL)	38.0 (29.0–46.0)
Causes of heart transplantation, no. (%)	
Ischemic	123 (19.8)
Cardiomyopathy	368 (59.4)
Valvular heart disease	25 (4.0)
Myocarditis	21 (3.4)
Infiltrative disease^a^	22 (3.5)
Congenital	21 (3.4)
Chemotherapy-induced	8 (1.3)
Panel-reactive antibody (PRA) > 50%	
Overall	124 (20.1)
Class-I	71 (11.6)
Class-II	85 (14.0)
Class-I & Class-II	37 (6.0)
Donor-specific antibodies (+)	74 (13.4)
Desensitization prior to HTx, no. (%)	50 (8.1)
Pre-operative support	
On IV inotropes	516 (83.2)
Mechanical support devices	
IABP	1 (0.2)
ECMO or PCPS	162 (26.1)
VAD	46 (7.4)
Ventilator	129 (20.8)
ECMO with ventilator	106 (17.1)
Operation time (min), median (IQR)	339.0 (286.0–405.0)
Cold ischemic time (min), median (IQR)	96.0 (65.0–169.0)
Warm ischemic time (min), median (IQR)	51.0 (38.0–75.0)
Post-op ECMO support, no. (%)	54 (8.7)
Post-op CRRT support, no. (%)	103 (16.6)
Donor characteristics	
Age (years), median (IQR)	43.0 (32.0–49.0)
Sex (male), no. (%)	442 (71.3)
BMI (kg/m^2^)	23.5 ± 3.6
Diabetes mellitus, no. (%)	31 (5.0)
Hypertension	88 (13.2)
LVEF (%)	63.2 ± 9.4
Total CPR time (min)	15.2 ± 24.7
Donor cause of death	
Intracranial hemorrhage	264 (42.6)
Trauma	156 (25.2)
Hanging	121 (19.5)
Other	79 (12.7)

Abbreviations: BMI, body mass index; BUN, blood urea nitrogen; CKD, chronic kidney disease; CPR, cardiopulmonary resuscitation; CRRT, continuous renal replacement therapy; ECMO, extracorporeal membrane oxygenation; eGFR, estimated glomerular filtration rate; HD, hemodialysis; HDL-C, high-density lipoprotein cholesterol; HTx, heart transplantation; IABP, Intra-aortic balloon pump; IQR, interquartile range; LDL-C, low-density lipoprotein cholesterol; VAD, ventricular assist device; PCPS; percutaneous cardiopulmonary support; WBC, white blood cell.

^a^ Infiltrative diseases including amyloidosis *n* = 8 (1.1%) and sarcoidosis *n* = 16 (2.1%).

The median donor age was 43.0 years (IQR 32.0–49.0), with males as the predominant sex (71.3%). The mean body mass index of the donors was 23.5 ± 3.6 kg/m^2^, and the mean LVEF was 63.2%. Donors with diabetes mellitus and hypertension accounted for 5.0% and 13.2% of all donors, respectively. The leading causes of donor death were intracranial hemorrhage (42.6%), trauma (25.2%), and suicide by hanging (19.5%). The mean cardiopulmonary resuscitation time was 15.2 ± 24.7 min.

### Immunosuppressive Agent Prescription Patterns

**FIGURE 1:**
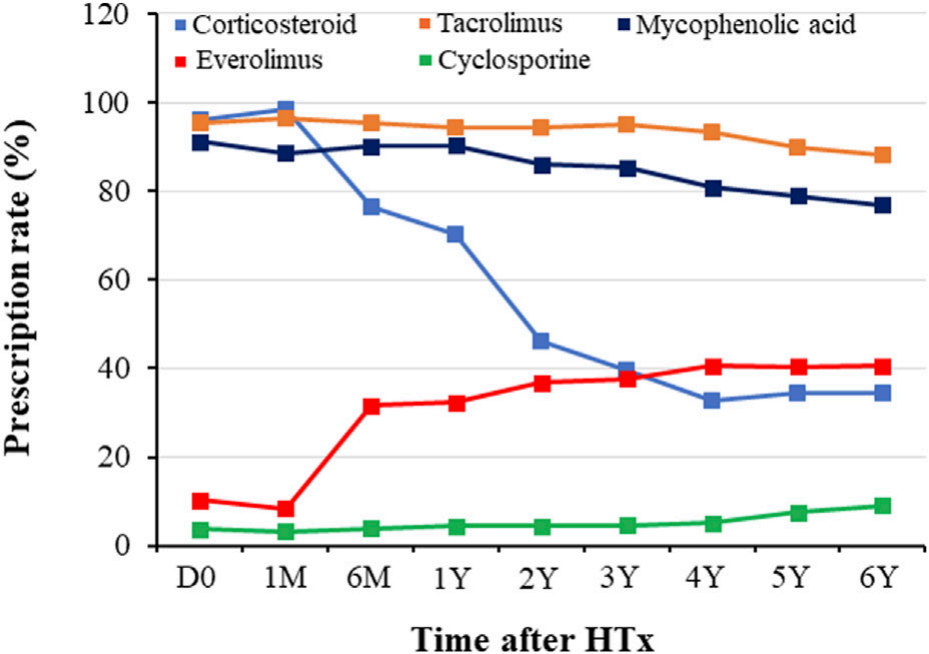
Temporal trend of immunosuppressant prescription during follow-up periods. Prescription patterns for immunosuppressants during the follow-up period. D0, discharge; HTx, heart transplantation; M, month; Y, year.

Prescription patterns for immunosuppressive agents during the follow-up period are shown in Figure 1. At discharge post-HTx, TAC, cyclosporine (CsA), MMF, CS, and everolimus (EVR) were prescribed to 95.5%, 3.8%, 91.1%, 96.2%, and 10.3% of recipients, respectively. The most frequently prescribed immunosuppressive agents were TAC (95.5% at discharge and 76.9% at the 6-year follow-up) and MMF (91.1% at discharge and 76.9% at 6-year follow-up). Notable changes in immunosuppressive agents were CS weaning (dose tapering or withdrawal) and EVR initiation. CS weaning attempts were initiated from the first month post-HTx, and the rate of CS prescription decreased from 96.2% at discharge to 34.3% at the 6-year follow-up. The prescription rate for EVR increased after the first month post-HTx, ranging from 10.3% at discharge to 31.6% at the 6-month follow-up and 40.6% at the 6-year follow-up. However, the CsA prescription rate was less than 5% during follow-up periods.

### Changes in Prescribed Immunosuppressive Agents and Maintenance Regimens

**FIGURE 2:**
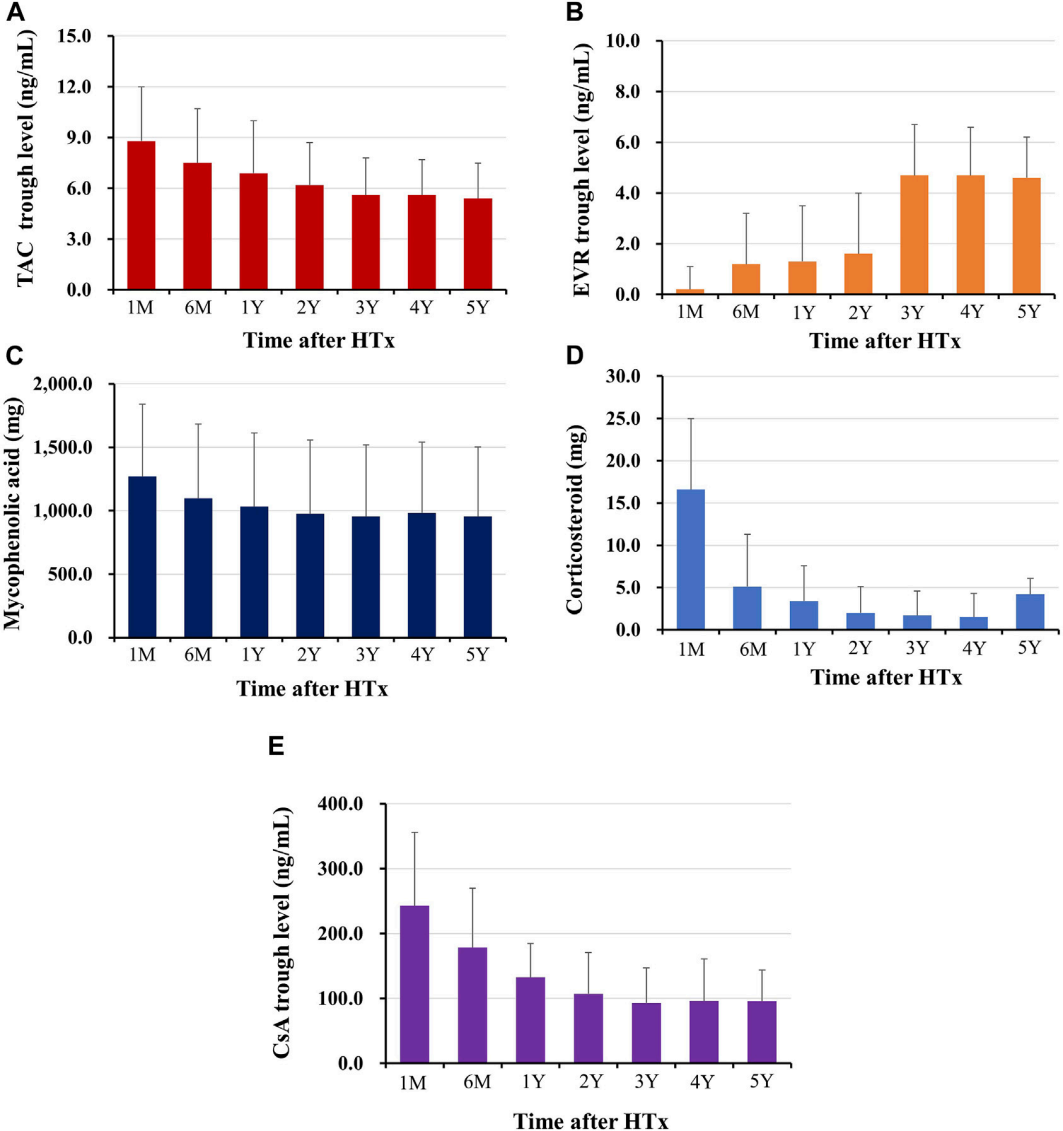
Changes in immunosuppressant prescription doses or trough levels during follow-up periods The trough level of tacrolimus **(A)**, and everolimus **(B)**, the prescription dose of mycophenolic acid **(C)**, and corticosteroid **(D)**, the trough level of cyclosporine **(E)** during the follow-up periods are shown. HTx, heart transplantation; M, month; Y, year.

**FIGURE 3:**
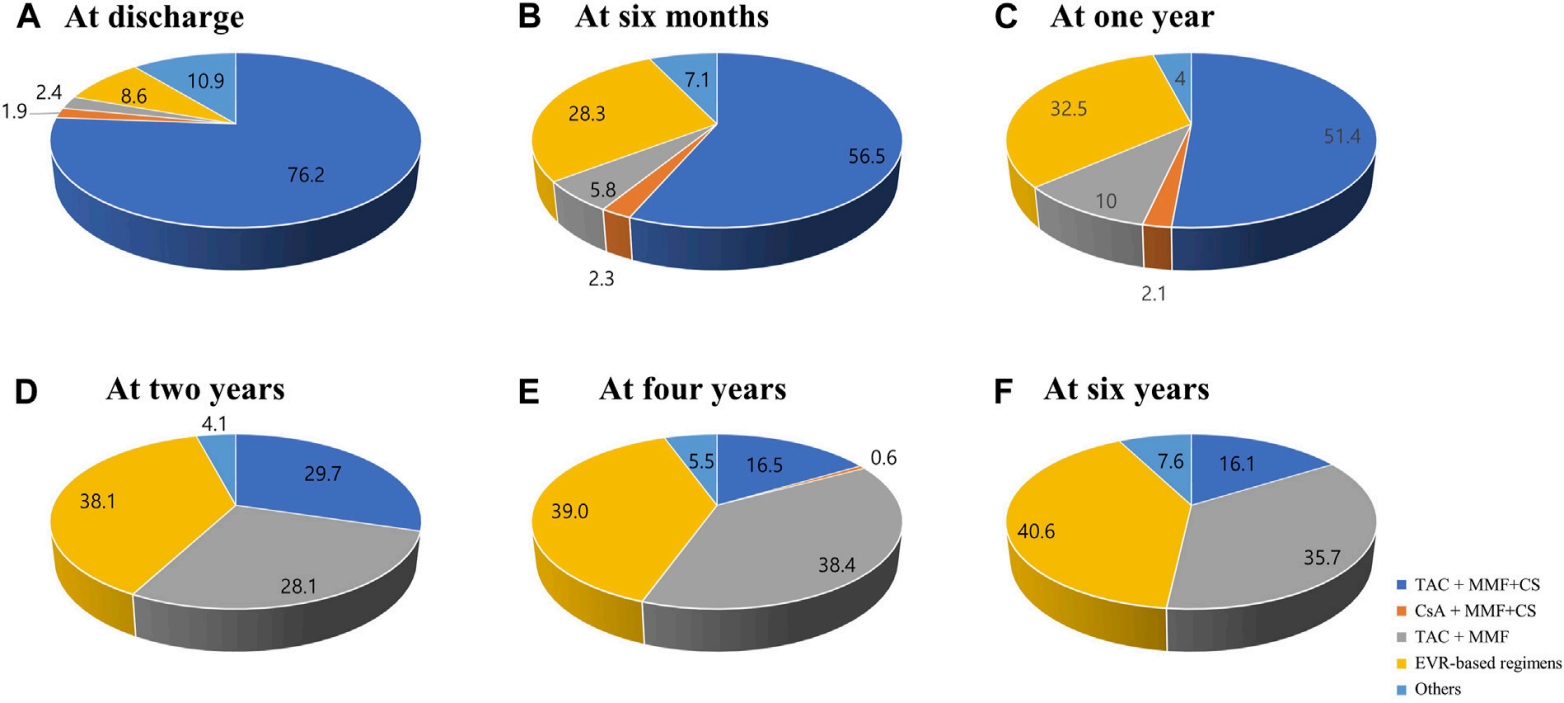
Changes in maintenance regimens post-HTx. The Venn diagram shows the changes in maintenance regimens at discharge **(A)**, 6 months **(B)**, 1 year **(C)**, 2 years **(D)**, 4 years **(E)**, and 6 years **(F)** post-HTx. CS, corticosteroid; CsA, cyclosporine; EVR, everolimus; HTx, heart transplantation; MMF, mycophenolate mofetil; TAC, tacrolimus.

The changes in prescribed immunosuppressive agent doses or in trough levels during the follow-up period are shown in Figure 2. The doses of immunosuppressive agents decreased rapidly in early post-HTx periods and remained constant throughout the follow-up period. The changes in maintenance regimens during follow-up period are shown in Figure 3. The most used maintenance regimen in the Korea during 2014–2021 was a triple therapy regimen consisting of TAC, MMF, and CS in the early phase post-HTx (Supplementary Figure S1). However, the prescription rate for TAC-based triple regimens gradually decreased from 76.2% at discharge to 16.1% at 6-year follow-up. The prescription rates for EVR-based regimens (from 8.6% at discharge to 40.6% at 6-year follow-up) and CS-free/TAC/MMF regimens (from 2.4% at discharge to 35.7% at 6-year follow-up) increased during follow-up periods (Figure 3). The EVR-based regimen consists of various combinations of immunosuppressive agents during the follow-up period. It consists of 4, 3, or 2 immunosuppressive agents including EVR (Supplementary Figure S2). Overall, the notable change in maintenance regimen was from TAC-based triple regimens to EVR-based or CS-free/TAC/MMF regimens via EVR initiation and CS weaning during follow-up periods.

**FIGURE 4:**
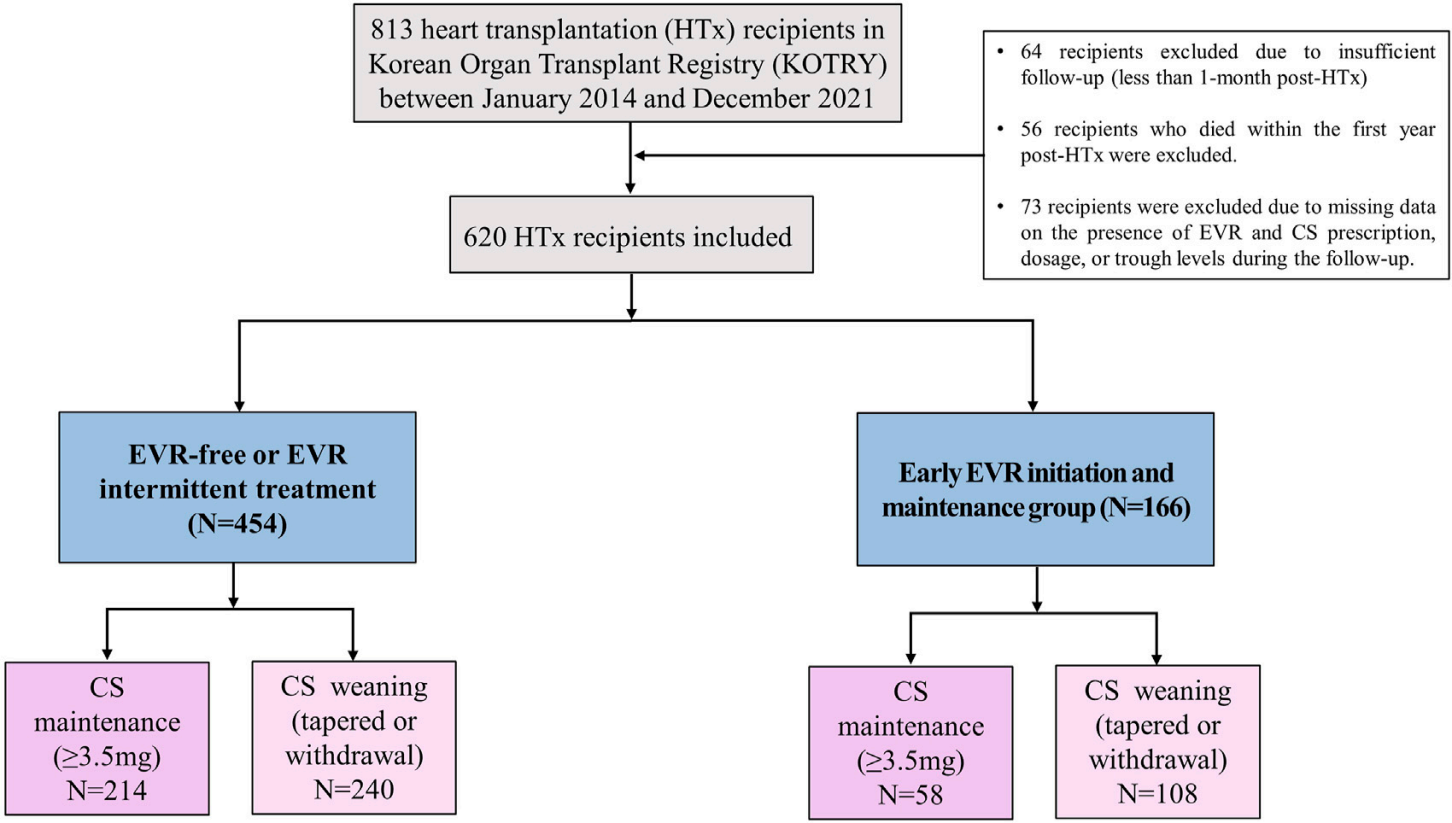
Study flow A flow chart of the selection of eligible HTx recipients for this study. CS, corticosteroid; EVR, everolimus; HTx, heart transplantation.

Based on these findings, we hypothesized that temporal changes in immunosuppressive regimens and the prescribed doses or trough levels in immunosuppressive agents during early post-HTx periods affect the clinical outcomes of recipients. Thus, a receiver operating characteristic (ROC) curve was used to assess the prognostic value of each immunosuppressive agent’s doses or trough levels during follow-up periods. We found that only CS and EVR doses or trough levels during the early post-HTx period (within the first year post-HTx) accurately predicted the primary outcome. ROC curves and optimal cutoff values of CS and EVR doses for the primary outcome are shown in Supplementary Figure S3. The CS dose at 1 year (AUC 0.72, sensitivity 64.7, specificity 58.3; *p* < 0.001) and EVR dose at 1 year (AUC 0.69, sensitivity 87.2, specificity 34.1; *p* < 0.001) showed good predictive ability for the primary composite outcome. The optimal cutoff values for predicting the primary outcome using the ROC curve and Youden index analyses were 3.5 mg (CS) and 0 mg (EVR). Based on these findings, HTx recipients (*n* = 620) were divided into CS weaning (CS withdrawal or tapered with less than 3.5 mg within the first year post-HTx) (*n* = 272) and CS maintenance (maintain CS more than 3.5 mg during the follow-up period) group (*n* = 346). In the case of EVR, the optimal cutoff value was 0 mg. For this reason, recipients were divided into EVR prescription and non-prescription groups. However, the treatment pattern of EVR in recipients was diverse in this study. Some patients were prescribed EVR intermittently, while others were continuously prescribed and taking EVR during the follow-up period. Therefore, HTx recipients were divided into three or two groups as follows: the EVR-free regimen group (*n* = 354), the EVR intermittent treatment regimen group (*n* = 100), and early EVR initiation/maintenance regimen group (*n* = 166) or EVR-free or EVR intermittent treatment regimen group (*n* = 454), and the early EVR initiation/maintenance regimen group (*n* = 166) (Figure 4).

### Clinical Outcomes

#### Primary Outcome

**FIGURE 5:**
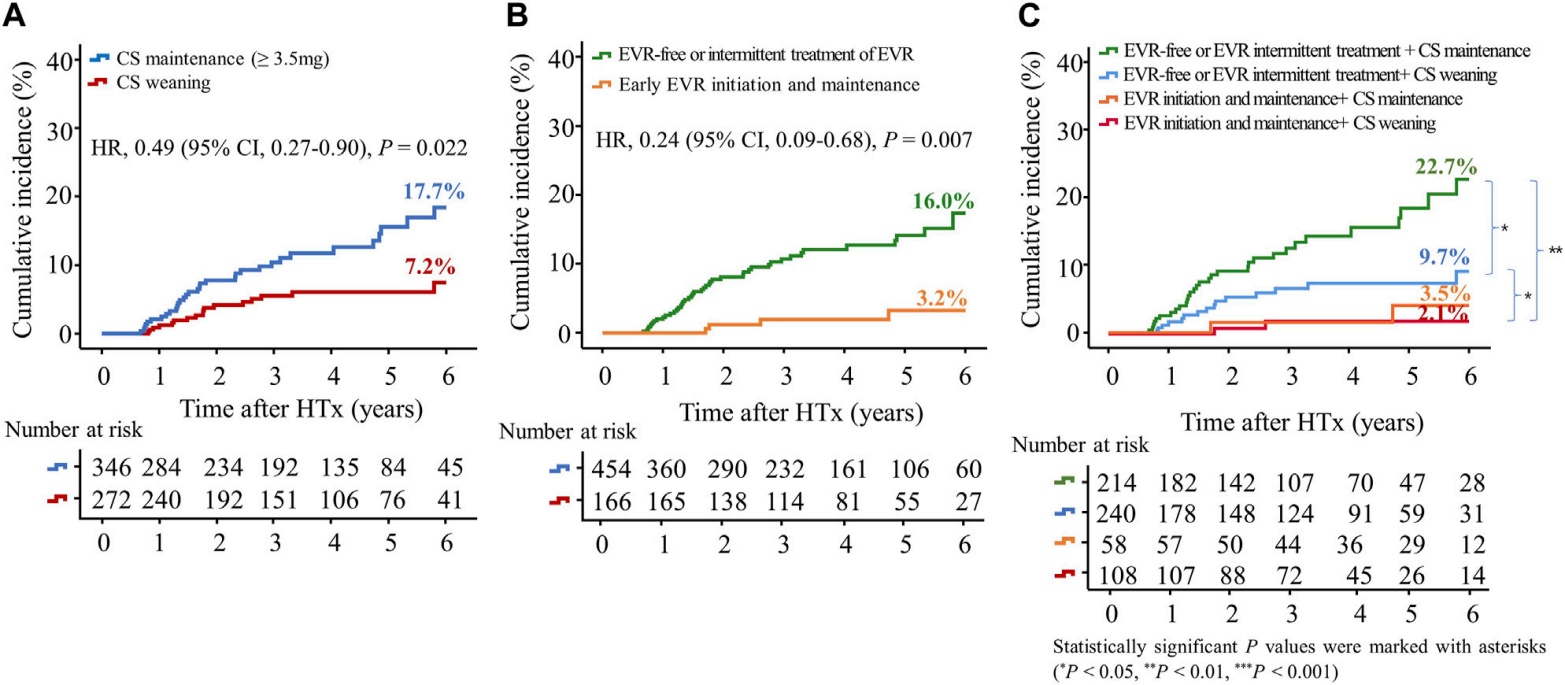
Impact of everolimus initiation and corticosteroid weaning on the primary outcome. The primary outcome was a composite of all causes of death or re-transplantation. Kaplan–Meier curves comparing the risk of primary outcome between the CS weaning (tapering (<3.5 mg) or withdrawal) and CS maintenance (≥3.5 mg) regimens **(A)** and between early EVR initiation and EVR-free regimens **(B)**. Kaplan–Meier curves comparing the risk of primary outcome between four groups according to CS weaning and the presence of EVR initiation **(C)**. CI, confidence interval; CS, corticosteroid; EVR, everolimus; HR, hazard ratio; HTx, heart transplantation.

To investigate the effects of early CS weaning during the first year post-HTx on clinical outcomes, we compared the clinical outcomes between the CS weaning within the first year post-HTx and CS dose maintenance (≥3.5 mg) during the follow-up. The early CS weaning within the first year post-HTx had reduced the risk of primary composite outcome (all-cause death or re-transplantation) compared with CS maintenance (≥3.5 mg) (7.2% vs. 17.7%; HR, 0.49; 95% CI 0.27–0.90, *p* = 0.022) (Figure 5A). Next, to investigate the effects of early EVR initiation within the first year post-HTx and continuously maintained EVR during the follow-up on clinical outcomes, we compared the clinical outcomes between the EVR-free or EVR intermittent treatment regimen group and the early EVR initiation/maintenance regimen group. The early EVR initiation during the first year post-HTx and continuously maintained EVR during the follow-up had reduced the risk of primary composite outcome compared with the EVR-free or EVR intermittent treatment regimen group (3.2% vs. 13.3% and 3.2% vs. 16.7%, log-rank *p* = 0.002 and *p* < 0.001, respectively). However, there was no significant difference in primary outcome between EVR-free and EVR intermittent treatment (Supplementary Figure S4). For this reason, EVR-free and EVR intermittent treatment regimen groups were combined and defined as an EVR-free or EVR intermittent treatment regimen group (Figure 4). Ultimately, to investigate the effect of early CS weaning and early EVR initiation/maintenance on clinical outcomes, 622 HTx recipients were divided into four subgroups as follows: early EVR initiation and maintenance with CS weaning regimen (*n* = 108), EVR-free or EVR intermittent treatment with CS weaning regimen (*n* = 240), early EVR initiation and maintenance with CS maintenance (≥3.5 mg) regimen (*n* = 58), and EVR-free or EVR intermittent treatment with CS maintenance regimen (*n* = 214).

The early EVR initiation during the first year post-HTx and continuously maintained EVR during the follow-up had reduced the risk of primary composite outcome compared with EVR-free or EVR intermittent treatment regimen group (3.2% vs. 16.0%; HR, 0.24; 95% CI 0.09–0.68, *p* = 0.007) regardless of CS weaning (Figures 5B, C). However, CS weaning within the first year post-HTx had reduced the risk of primary composite outcome compared with CS maintenance (≥3.5 mg) in EVR-free or EVR intermittent treatment regimen (9.7% vs. 22.7%; HR, 0.51; 95% CI 0.27–0.97, *p* = 0.042) (Figure 5C). An EVR-based regimen had reduced the risk of primary composite outcome compared with the TAC + MMF + CS regimen (HR, 0.41; 95% CI 0.17–0.99, *p* = 0.048) and other regimens (HR, 0.21; 95% CI 0.08–0.56, *p* = 0.002) (Supplementary Figure S5A).

#### Cardiac Allograft Vasculopathy

**FIGURE 6:**
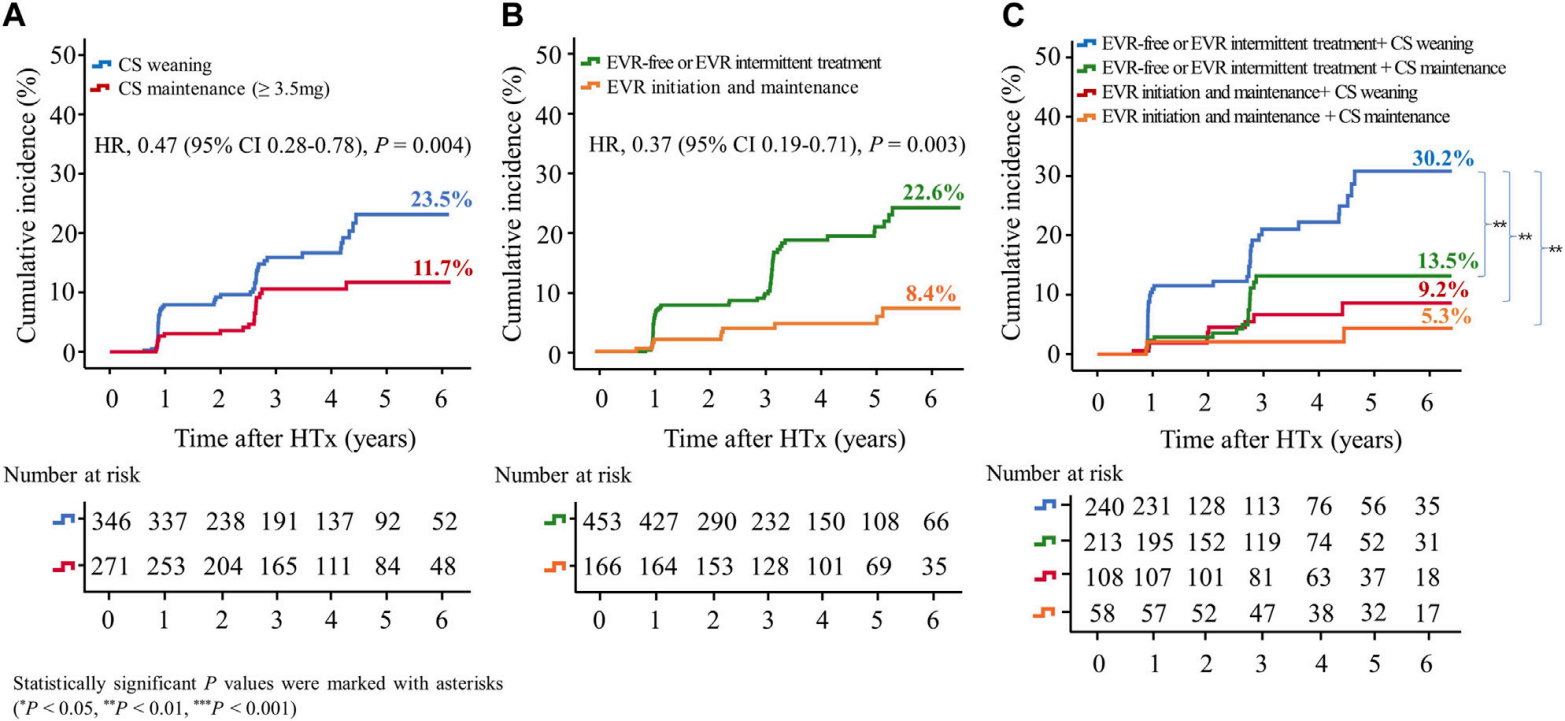
Impact of everolimus initiation and corticosteroid tapering or withdrawal on CAV. The Kaplan–Meier curve shows the cumulative incidence of CAV. The cumulative incidence of CAV significantly decreased in HTx recipients undergoing CS maintenance (≥3.5 mg) compared with those undergoing CS tapering (<3.5 mg) or withdrawal regimens **(A)**, and in recipients undergoing early EVR initiation compared with those undergoing EVR-free regimens **(B)**. The cumulative incidence of CAV is the highest in HTx patients undergoing CS tapering (<3.5 mg) or withdrawal and EVR-free regimens compared with those undergoing other regimens **(C)**. CAV, cardiac allograft vasculopathy; CS, corticosteroid; EVR, everolimus; HR, hazard ratio; HTx, heart transplantation.

In this study, CAV events were identified in the first year after HTx, and CAV grades were mostly mild to moderate (Supplementary Table S1). The cumulative incidences of CAV according to CS weaning and the presence of EVR initiation are shown in Figure 6. The incidence of CAV decreased in the CS maintenance group compared with in the CS weaning group (11.7% vs. 23.5%, HR 0.47; 95% CI 0.28–0.78, *p* = 0.004) and in the EVR initiation/maintenance regimen group compared with in the EVR-free or intermittent EVR treatment regimen group (8.4% vs. 22.6%, HR 0.39; 95% CI 0.19–0.79, *p* = 0.009), respectively (Figures 6A,B). Furthermore, CS maintenance (≥3.5 mg) reduced the risk of CAV events compared with CS weaning in the EVR-free or intermittent treatment regimen group (13.5% vs. 30.2%, HR 0.44; 95% CI 0.25–0.77, *p* = 0.004) (Figure 6C).

The cumulative Incidence of CAV decreased in the EVR-based regimen group (7.8% vs. 24.2%, HR, 0.34; 95% CI, 0.17–0.68, *p* = 0.002) compared with the TAC + MMF + CS regimen group (Supplementary Figure S5B).

#### Acute Rejection

**FIGURE 7:**
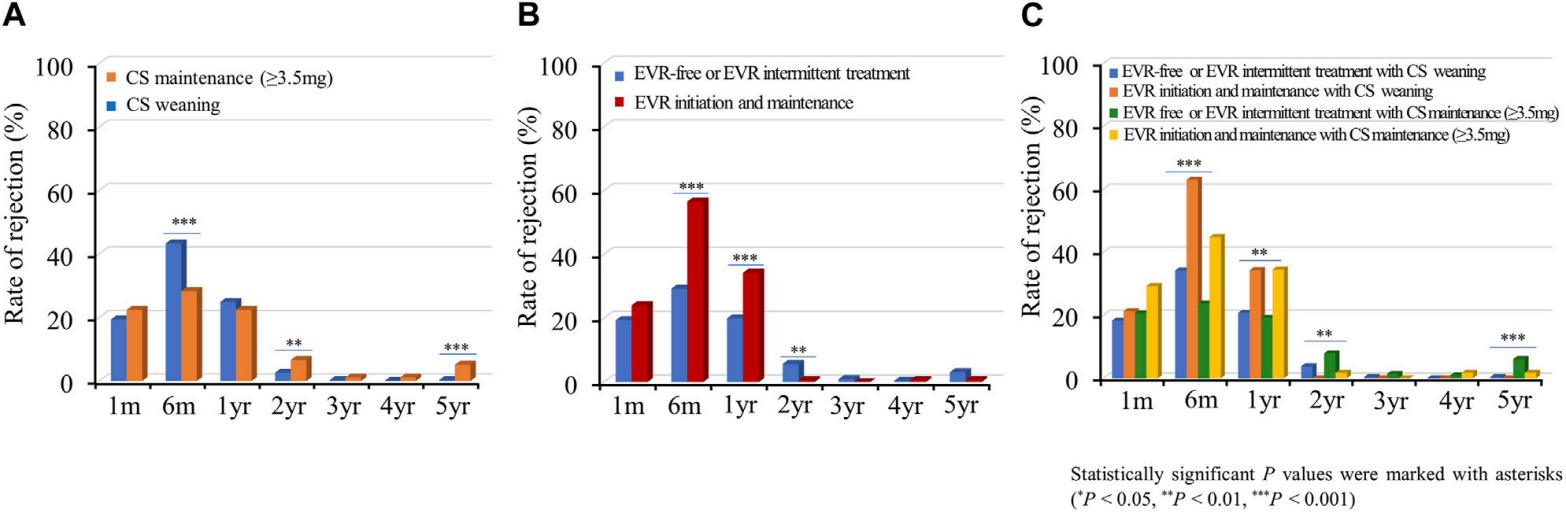
Impact of everolimus initiation and corticosteroid weaning on acute cellular rejection. The rate of acute cellular rejection is shown according to the CS weaning **(A)**, EVR initiation **(B)**, and the combination of EVR initiation and CS weaning **(C)** regimens during the follow-up period. CS, corticosteroid; EVR, everolimus, HTx, heart transplantation.

Acute allograft rejection occurred most frequently during the first 6 months post-HTx, and the number of rejections decreased during the follow-up periods (Figures 7A–C; Supplementary Figure S6). Rates of biopsy-proven acute cellular rejection (ACR) and acute antibody-mediated rejection (AMR) were higher in the CS weaning group at 6 months and at 1 month post-HTx than in the CS maintenance group (Figure 7A; Supplementary Figure S6A). Furthermore, biopsy-proven ACR and AMR were higher in the early EVR initiation/maintenance regimen group during the first year post-HTx than in the EVR-free or EVR intermittent treatment regimen group (Figures 7B, C; Supplementary Figure S6).

#### Infection and Malignancy

**FIGURE 8:**
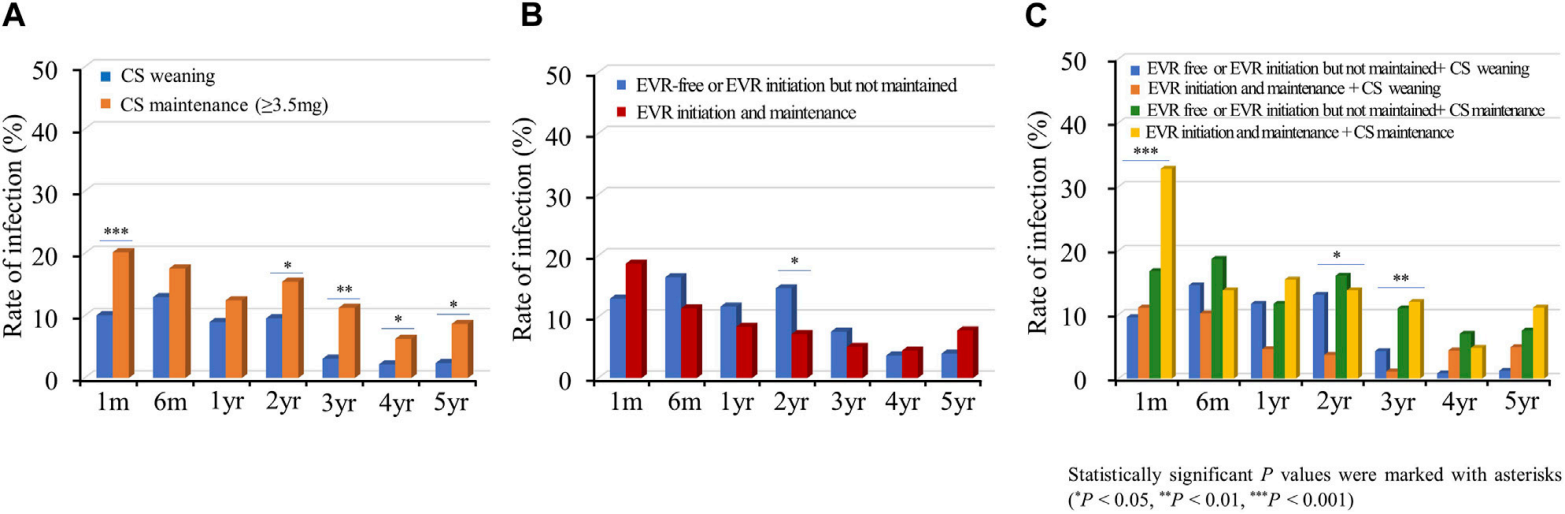
Impact of everolimus initiation and corticosteroid weaning on infection. The incidence of infection requiring hospitalization is shown according to the CS weaning **(A)**, EVR initiation **(B)**, and the combination of EVR initiation and CS weaning **(C)** regimens during the follow-up period. CS, corticosteroid; EVR, everolimus; HTx, heart transplantation.

Subsequently, we investigated the rate of infections requiring hospitalization according to the immunosuppressive regimen. Infection events frequently occurred in the acute phase post-HTx, and the infection rate decreased during the follow-up period (Figure 8). The CS maintenance (≥3.5 mg) significantly increased the incidence of infection during the follow-up period (Figure 8A). No significant difference was found in rates of infections requiring hospitalization between the EVR initiation/maintenance regimen group and the EVR-free or EVR intermittent treatment regimen group except for the 2-year follow-up period (Figure 8B). However, the CS maintenance (≥3.5 mg) or EVR initiation/maintenance with CS maintenance (≥3.5 mg) regimens were associated with higher risks of infection compared with other regimens during the follow-up period (Figure 8C).

The incidence of malignancies tends to slightly increase during the follow-up period. The CS maintenance (≥3.5 mg) slightly increased the incidence of malignancy during the follow-up period compared with CS weaning (8.5% vs. 4.7%; OR, 1.7; 95% CI 0.9–3.4, *p* = 0.125). However, there was no significant difference in the incidence of malignancy between the early EVR initiation/maintenance and EVR-free or intermittent treatment (6.6% vs. 6.4%; OR, 1.0; 95% CI 0.5–2.1, *P* = 0.973). The EVR initiation and maintenance with CS maintenance regimen tend to increase the risk of malignancy compared to EVR initiation and maintenance with CS weaning regimen (12.1% vs. 3.7%; OR, 3.0; 95% CI 0.8–11.0, *p* = 0.105) (Supplementary Table S2; Supplementary Figure S7–S9).

## Discussion

This study was the first to investigate changes in the immunosuppressive agents and maintenance regimens and evaluated the effects of these temporal changes during the acute phase on the clinical outcomes in Korean HTx recipients. The major characteristics of the immunosuppressive agents for HTx recipients enrolled in the KOTRY were as follows: First, the initial backbone of immunosuppressive maintenance regimens was TAC and MMF. Second, notable changes in the prescription of CS and EVR were found. CS weaning attempts were initiated after one-month post-HTx, and the rate of CS prescription and dose gradually decreased during the follow-up period. However, the prescription of EVR increased after one-month post-HTx. Third, temporal dose changes in immunosuppressive agents mainly occurred during the acute phase (within 1 year post-HTx). Fourth, the maintenance immunosuppressive therapy was changed from a TAC-based triple therapy (TAC + MMF + CS) to EVR-based and CS-free/TAC/MMF therapy during the follow-up period.

CS are important components of induction, maintenance, and rejection regimens post-HTx [2, 6]; however, CS administration was associated with the highest number of long-term adverse effects. Therefore, attempts at CS withdrawal or dose tapering are continuously being made in the HTx field. Delgado et al. [23] reported that the use of CS for more than 1 year post-HTx is unlikely to provide clinical benefits. Furthermore, the ISHLT guidelines recommend that CS withdrawal can be achieved within 3–12 months post-HTx in low-rejection risk patients to minimize CS adverse effects [21]. Consistent with other studies, our study showed that CS weaning within the first year post-HTx was associated with a reduced risk of the primary outcome. However, the effects of CS weaning on the primary outcome differed according to the presence of early EVR initiation. In EVR-free or EVR intermittent treatment regimens, CS dose maintenance (≥3.5 mg) had a higher risk of the primary composite outcome than CS weaning. However, no significant difference was observed in the primary composite outcome between the two groups in the early EVR initiation/maintenance regimen. This may be explained by the effect of EVR. Recipients receiving EVR during the follow-up period had a lower mean CS dose compared with recipients who were not administered EVR (Table 2). For this reason, adverse effects due to CS dose maintenance during the follow-up period would have been minimized.

**TABLE 2:** Changes in immunosuppressive agent dosages or trough levels according to the presence of everolimus prescription post-HTx.

	EVR-free or EVR intermittent treatment	Early EVR initiation and maintenance	*p*-Value
Discharge after HTx			
TAC (ng/mL)^a^	5.1 ± 3.6	5.4 ± 3.7	0.665
CsA (ng/mL)^a^	196.7 ± 89.6	203.3 ± 116.0	0.889
MMF (mg)	1,278.3 ± 559.0	943.1 ± 519.4	<0.001
CS (mg)	18.2 ± 12.8	11.0 ± 7.2	<0.001
One month after HTx			
TAC (ng/mL)	9.2 ± 3.3	8.1 ± 2.6	<0.001
CsA (ng/mL)	232.9 ± 100.8	227.7 ± 121.4	0.915
MMF (mg)	1,309.6 ± 663.3	797.9 ± 435.3	<0.001
CS (mg)	16.9 ± 8.4	13.6 ± 7.5	0.004
Six months after HTx			
TAC (ng/mL)	8.2 ± 3.1	5.6 ± 2.5	<0.001
CsA (ng/mL)	192.4 ± 67.6	147.0 ± 67.6	0.182
MMF (mg)	1,285.1 ± 562.8	612.6 ± 289.6	<0.001
CS (mg)	6.2 ± 6.8	2.7 ± 3.5	<0.001
One year after HTx			
TAC (ng/mL)	7.7 ± 3.2	4.8 ± 1.7	<0.001
CsA (ng/mL)	141.2 ± 56.9	119.1 ± 42.7	0.280
MMF (mg)	1,207.7 ± 579.0	606.1 ± 294.6	<0.001
CS (mg)	4.0 ± 4.5	2.1 ± 3.1	<0.001
Two years after HTx			
TAC (ng/mL)	7.4 ± 1.9	4.5 ± 1.6	<0.001
CsA (ng/mL)	121.3 ± 71.9	84.7 ± 42.6	0.070
MMF (mg)	1,134.7 ± 582.4	597.5 ± 371.0	<0.001
CS (mg)	2.1 ± 2.8	1.7 ± 3.6	0.126
Three years after HTx			
TAC (ng/mL)	6.4 ± 2.1	4.0 ± 1.3	<0.001
CsA (ng/mL	117.9 ± 56.5	65.7 ± 35.7	0.029
MMF (mg)	1,097.8 ± 576.2	607.8 ± 351.9	<0.001
CS (mg)	1.9 ± 3.1	1.4 ± 2.3	0.098
Four years after HTx			
TAC (ng/mL)	6.4 ± 2.0	4.0 ± 1.1	<0.001
CsA (ng/mL	127.1 ± 76.0	61.3 ± 21.9	0.032
MMF (mg)	1,139.2 ± 571.3	617.6 ± 299.6	<0.001
CS (mg)	1.6 ± 3.1	1.3 ± 2.3	0.380

Abbreviations: CS, corticosteroid; CsA, cyclosporine; EVR, everolimus; HTx, heart transplantation; MMF, mycophenolic mofetil; TAC, tacrolimus.

^a^ TAC and CsA were represented to trough level.

However, it is possible that the initiation of EVR and CS weaning attempts were preferentially considered in recipients at low risk of rejection. Conversely, recipients at high risk of rejection may have been maintained on a higher dose of CS. Therefore, considering the confounding and selection bias, we should be cautious in extrapolating the current results to all HTx recipients who may have a different immunosuppressant regimen. This study showed that various EVR-based regimens are being applied in HTx recipients. Furthermore, the EVR initiation is associated with changes in prescription rate or dose of other immunosuppressive agents including TAC, MMF, or CS. Because the initiation of EVR indirectly affects changes in the prescription rate or dosage of other immunosuppressive agents, further research is needed to confirm whether our results are a direct effect on EVR or an effect due to changes in the prescription rate or dose of other immunosuppressive agents.

The safety and efficacy of mTOR inhibitor treatment have been reported [11, 12]. The SCHEDULE study showed that the EVR initiation with cyclosporine withdrawal 7–11 weeks after HTx reduced CAV progression at 12 months than standard cyclosporine-based immunosuppression [11]. Furthermore, early conversion (median time of 1.1 years [IQR 0.6–3.0 years]) to sirolimus is associated with attenuated CAV progression, lower long-term mortality, and fewer CVA-related events than continued CNI use [12]. However, the EVERHEART study reported that the initiation of mTOR inhibitors immediately (≤144 h post-HTx) post-transplantation is associated with a poor safety profile, driven primarily by a higher rate of pericardial effusions compared with delayed (4–6 weeks post-transplantation) mTOR inhibitor treatment initiation [13]. These varying results suggest that the optimal timing of mTOR inhibitor treatment initiation to maintain an adequate balance between drug efficacy and safety remains unclear.

In this study, the early (within the first year post-HTx) EVR initiation and maintenance during the follow-up period is associated with reduced risk of the primary composite outcome and CAV events in recipients compared with the EVR-free or intermittent treatment regimens. Furthermore, compared with the TAC-based triple regimen (TAC + MMF + CS), the EVR-based regimen is associated with reduced risk of the primary composite outcome and CAV events. These results suggest that the early EVR initiation-based regimen can be an alternative treatment option for HTx recipients to improve clinical outcomes. The EVR-based regimen largely consists of a combination of 4, 3, or 2 immunosuppressants including EVR during the follow-up period. In clinical settings, EVR is used for HTx recipients for a variety of reasons. Although the reason for switching or adding EVR from other immunosuppressants is not clearly described in the KOTRY heart transplant cohort, the use of EVR is primarily [1] to increase immunosuppression in the early phase after HTx [2], for minimization of other immunosuppressants (TAC, MMF or CS) or for CNI-free regimen, and [3] when CAV is suspected. In our study, the prescription rates, doses, or trough levels of TAC, CsA (cyclosporine A), MMF, or CS are lower in the early EVR initiation and maintenance regimen than in the EVR-free or EVR intermittent treatment regimen (Table 2; Supplementary Table S3). Furthermore, the TAC trough level is significantly lower in the early EVR initiation and maintenance regimen than in the EVR-free or EVR intermittent treatment regimen (Supplementary Table S4). These findings suggest that the use of EVR is associated with the minimization of other immunosuppressive agents or conversion to a CNI-free regimen.

In the EVR-free or EVR intermittent treatment regimen, TAC trough levels are lower in the third (Q3) or fourth (Q4) quartiles than in the lower quartiles (Q1 and Q2) of the serum creatinine during the follow-up period. However, there was no significant difference in TAC trough levels between lower quartiles (Q1 and Q2) and the third (Q3) or fourth (Q4) quartiles of the serum creatinine in the early EVR initiation and maintenance regimen (Supplementary Table S4). Furthermore, TAC trough levels and serum creatinine levels were lower in the early EVR initiation and maintenance regimen than in the EVR-free or EVR intermittent treatment regimen (Supplementary Tables S4, S5). These findings suggest that the initiation of EVR may not affected by serum creatinine levels. However, the early initiation of EVR is associated with a reduced risk of CNI-related nephrotoxicity by minimizing CNI exposure during the follow-up period.

EVR is a mammalian target of rapamycin (mTOR) inhibitor/proliferation-signal inhibitor with potent immunosuppressive and anti-proliferative effects. Several studies have demonstrated the efficacy of EVR in reducing acute rejection, progression, and development of CAV [11, 24]. Furthermore, EVR has the potential to facilitate the reduction of CNI therapy and preserved renal function [10, 15]. The current study’s findings on the efficacy of EVR initiation were consistent with previous studies. However, tolerability and safety of EVR remain a concern. EVR-related pneumonitis, pericardial effusion, mouth ulcers, and impaired wound healing were associated with morbidity and mortality. Another issue is which immunosuppressive agents should be used in combination with EVR in HTx recipient during the long-term period. The combination of EVR and CS may be associated with a reduced risk of rejection and the progression or development of CAV by enhancing immunosuppression in HTx recipients. Although there are limitations in drawing conclusions due to the number of subjects in this study being relatively small, long-term treatment of EVR and CS combination therapy may have increased the incidence of infection or malignancy compared to EVR with CS weaning therapy in our study (Supplementary Table S2; Figure 8; Supplementary Figure S9). Considering that our study is an observational study, and the sample size is small, further studies are needed to verify the safety of long-term treatment of EVR and CS combination therapy.

CAV remains a long-term complication of HTx and is the major cause of death in patients surviving 1 year after transplantation [3, 25, 26]. According to a previous study, the prevalence of CAV is 3.3%, 5.1%, and 9.7% at one, two, and 5 years after transplantation, respectively [27]. The occurrence of CAV in our study was confirmed in the first year after transplantation, and the incidence rates were 5.5%, 6.1%, 12.3%, 13.7%, 15.4%, and 14.7% at one, two, three, four, five, and 6 years after transplantation, respectively. Although the grade of CAV was mostly mild (CAV 1) to moderate (CAV 2), an early EVR initiation-based regimen effectively prevented CAV progression. Furthermore, CS prevented CAV progression in recipients receiving EVR-free or EVR intermittent treatment regimens in our study. CS and EVR had a synergistic effect in preventing CAV. CAV incidence was the highest in EVR-free or EVR intermittent treatment with CS weaning regimen, whereas CAV incidence was the lowest in the early EVR initiation/maintenance with CS maintenance regimen (30.2% vs. 5.3%, *p* = 0.002). However, even if CS prevents CAV progression, CS is not effective in terms of CAV prevention considering the adverse effects that may occur due to long-term CS administration.

These findings suggest that the early initiation of EVR and maintenance therapy post-HTx may be reasonable, considering the efficacy of EVR. However, although the intention of the early initiation of EVR during the first year post-HTx is to effectively suppress immunity in recipients at high risk of rejection, the early EVR initiation may increase the risk of acute rejection due to reduced prescribed doses or trough levels of other immunosuppressive agents, including TAC, MMF, or CS. This finding suggests that changes in regimen, dose, or trough level of immunosuppressive agents during the first year post-HTx, when the risk of acute allograft rejection is the highest, may increase the risk of acute rejection. Therefore, these changes can increase the risk of acute rejection by destabilizing the patient’s immunosuppressive state during the first year post-HTx. The KOTRY data revealed that the prescription rates for TAC and MMF were consistently higher than those for other immunosuppressive agents during the follow-up period. At 6 years post-HTx, TAC and MMF prescription rates were 88.1% and 76.9%, respectively, with 35.7% of patients prescribed a TAC/MMF regimen and 16.1% of patients prescribed a TAC + MMF + CS regimen. Despite the TAC-based regimen increasing the risk of primary composite outcome and the incidence of CAV compared with the EVR-based regimen, 51.8% of recipients in Korea were still prescribed a TAC/MMF-based regimen. CS withdrawal was 65.6% at the 6-year follow-up post-HTx, whereas the prescription rate for EVR rapidly increased from 8.1% to 31.6% between one and 6 months but slightly increased thereafter to 40.6% at the 6-year follow-up. Although the excellent efficacy of EVR has been demonstrated in trials, several possible reasons exist for the low prevalence of early EVR-based regimens in Korea. First, the adverse effects and lower tolerability of EVR may affect their early or long-term use in HTx recipients. Second, adherence to traditional TAC-based regimens limits the use of EVR. Additional clinical studies are needed to investigate the use of early EVR-based maintenance regimens as an effective treatment strategy for HTx recipients.

This study has several limitations. First, this study was a retrospective observational study, and the analysis was based on a heart transplant cohort in KOTRY which has not been externally validated. Therefore, the results should be generalized with caution. Second, potential confounding and selection bias regarding CS weaning and EVR initiation may exist in a selected group of recipients. Further, we excluded 56 patients that died within the first year post-HTx due to evaluate the effect of CS weaning and EVR initiation during the first year post-HTx on long term clinical outcome. However, this exclusion may influence outcomes. Third, the indication and timing of CS weaning and EVR initiation differed per patient in the KOTRY. This is likely influenced by center-specific protocols and physician expertise or recipient characteristics and tolerability. This raises a very important bias (confounding by indication). Fourth, some information on the prescription status, dose, or trough level of immunosuppressive agents is missing during the follow-up. Finally, this study was conducted on an Asian population. Therefore, caution should be exercised when extrapolating these results to non-Asian HTx recipients.

In conclusion, the early EVR initiation within the first year post-HTx and maintenance during the follow-up period is associated with reduced risk of primary composite outcome and CAV events in HTx recipients. However, changes in the prescription rate, dose, or trough level of TAC, MMF, or CS due to early EVR initiation may increase the risk of acute allograft rejection during the first year post-HTx.

## Data Availability

The raw data supporting the conclusion of this article will be made available by the authors, without undue reservation.

## Footnotes

1
https://www.kotry.org/ko/main.html

## Abbreviations

Anti-HLA, antibodies of human leukocyte antigen; CAV, cardiac allograft vasculopathy; CI, confidence interval; CNI, calcineurin inhibitor; CS, corticosteroid; HR, hazard ratio; HTx, heart transplantation; ISHLT, International Society for Heart and Lung Transplantation; KOTRY, Korean Organ Transplant Registry; LVEF, left ventricular ejection fraction; MMF, mycophenolate mofetil; EVR, everolimus; TAC, tacrolimus; VA-ECMO, venoarterial extracorporeal membrane oxygenation.
