# Supplementary material for: Impact of Everolimus Initiation and Corticosteroid Weaning During Acute Phase After Heart Transplantation on Clinical Outcome: Data from the Korean Organ Transplant Registry (KOTRY)
Source: Transpl Int. 2024 Apr 5;37:11878. doi: 10.3389/ti.2024.11878 (PMC11028401; doi:10.3389/ti.2024.11878)
Supplement: Supplementary file 1 [file Presentation1.pptx]

## Slide 1
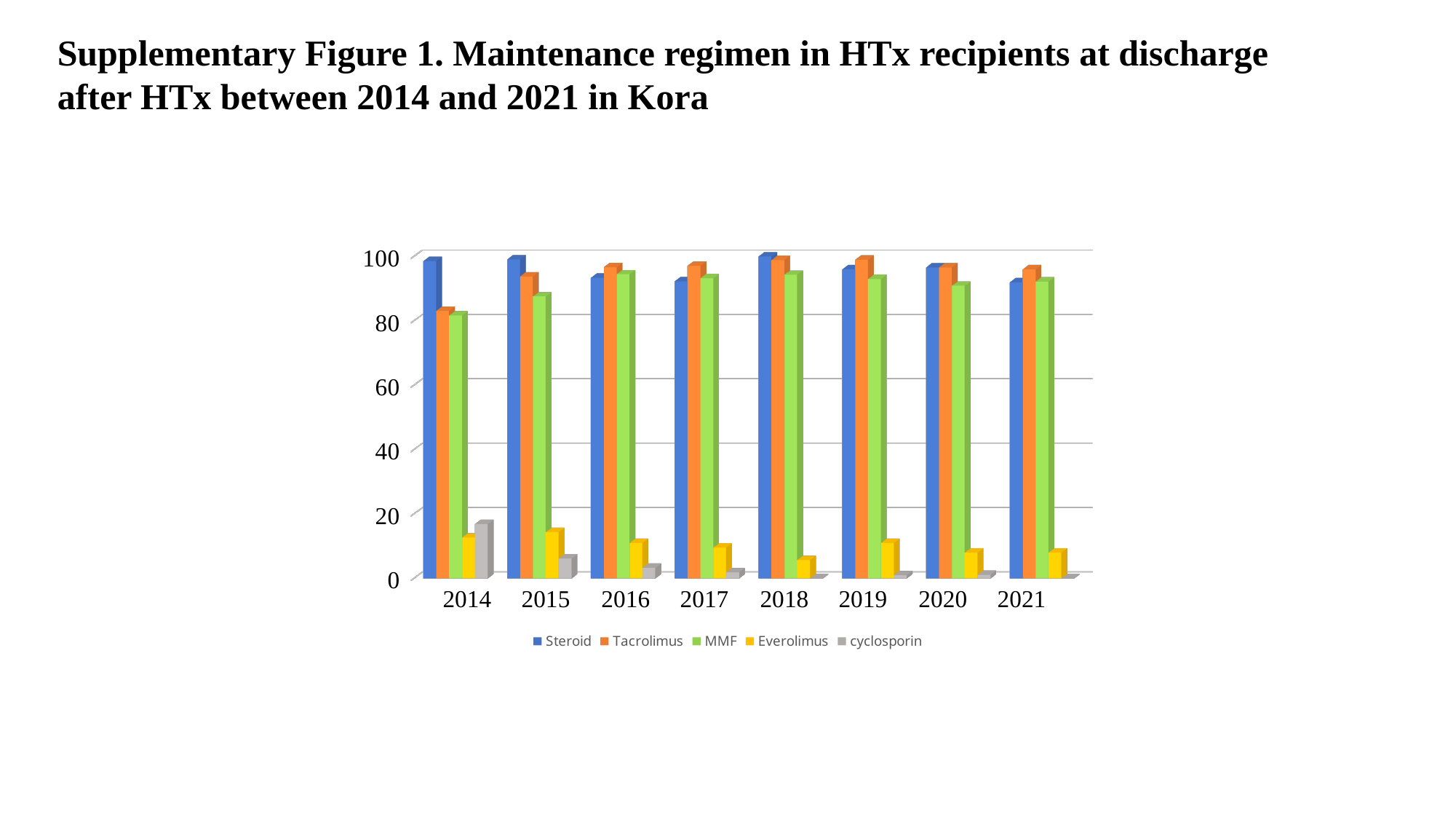

Supplementary Figure 1. Maintenance regimen in HTx recipients at discharge
after HTx between 2014 and 2021 in Kora
[unsupported chart]
2014
2015
2016
2017
2018
2019
2020
2021

## Slide 2
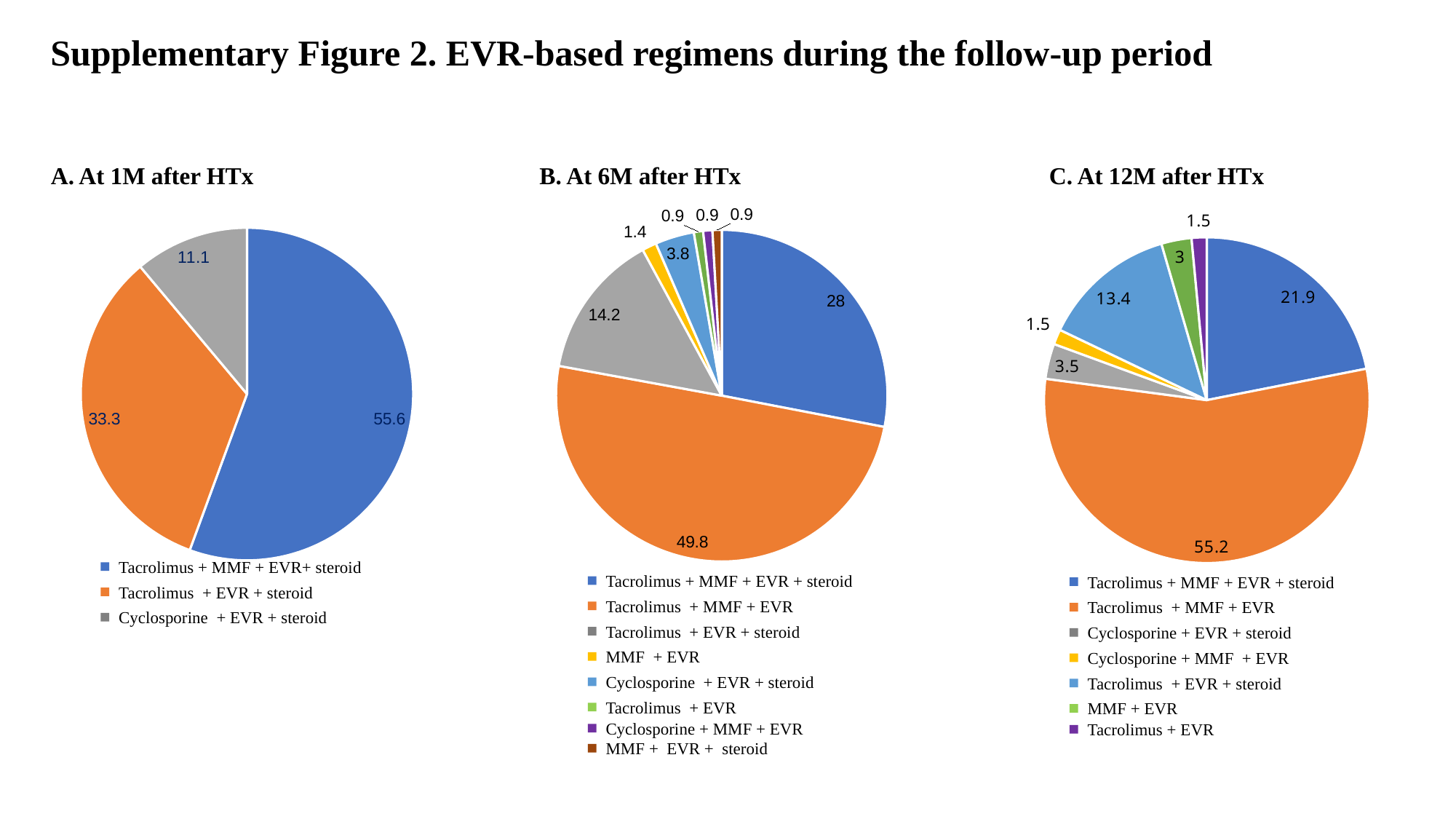

Supplementary Figure 2. EVR-based regimens during the follow-up period
A. At 1M after HTx
B. At 6M after HTx
C. At 12M after HTx
### Chart
| Category | |
|---|---|
### Chart
| Category | | |
|---|---|---|
### Chart
| Category | |
|---|---|Tacrolimus + MMF + EVR+ steroid
Tacrolimus + EVR + steroid
Cyclosporine + EVR + steroid
Tacrolimus + MMF + EVR + steroid
Tacrolimus + MMF + EVR
Tacrolimus + EVR + steroid
MMF + EVR
Cyclosporine + EVR + steroid
Tacrolimus + EVR
Cyclosporine + MMF + EVR
MMF + EVR + steroid
Tacrolimus + MMF + EVR + steroid
Tacrolimus + MMF + EVR
Cyclosporine + EVR + steroid
Cyclosporine + MMF + EVR
Tacrolimus + EVR + steroid
MMF + EVR
Tacrolimus + EVR

## Slide 3
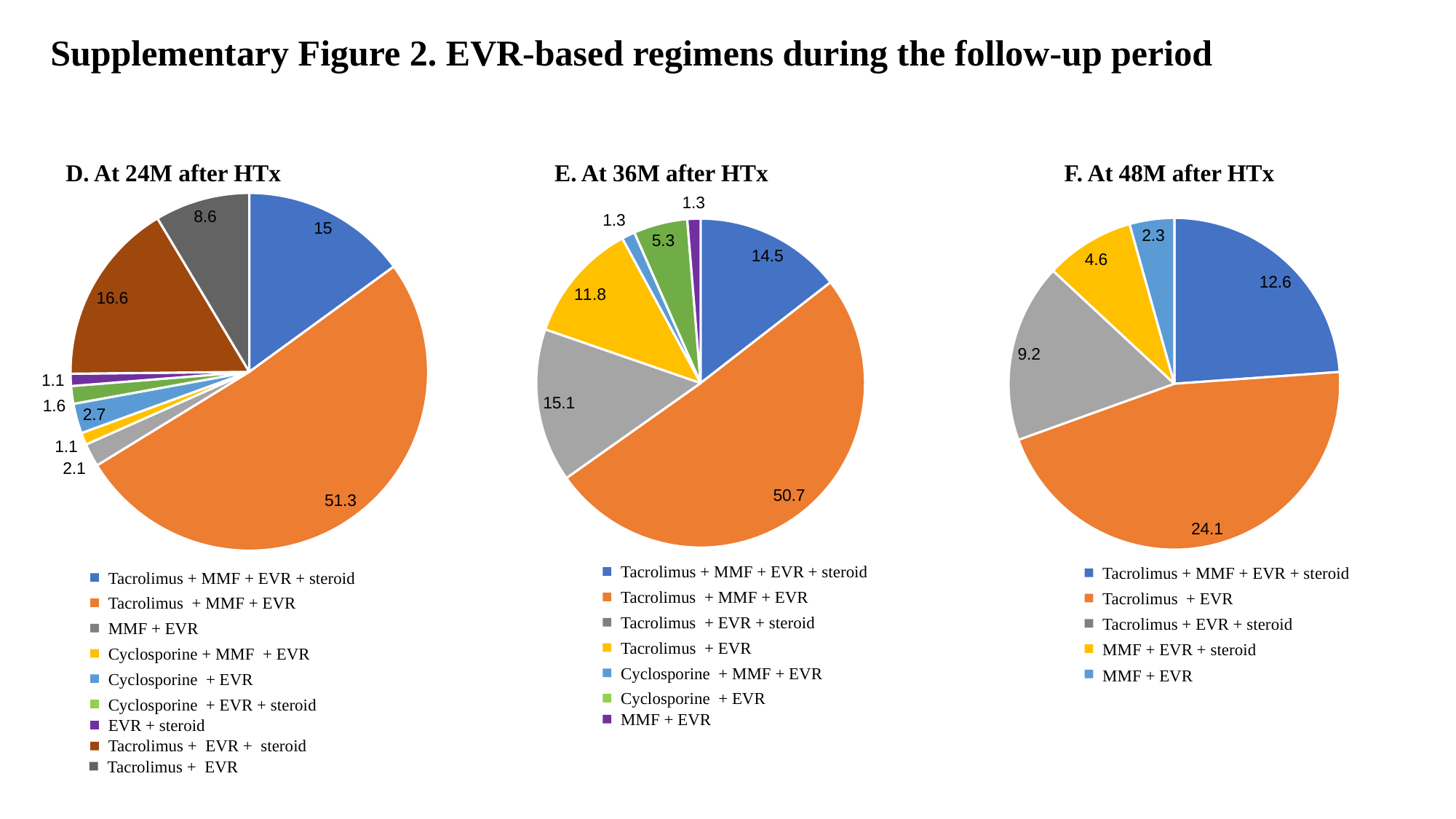

Supplementary Figure 2. EVR-based regimens during the follow-up period
D. At 24M after HTx
E. At 36M after HTx
F. At 48M after HTx
### Chart
| Category | |
|---|---|
### Chart
| Category | |
|---|---|
### Chart
| Category | |
|---|---|Tacrolimus + MMF + EVR + steroid
Tacrolimus + MMF + EVR
Tacrolimus + EVR + steroid
Tacrolimus + EVR
Cyclosporine + MMF + EVR
Cyclosporine + EVR
MMF + EVR
Tacrolimus + MMF + EVR + steroid
Tacrolimus + EVR
Tacrolimus + EVR + steroid
MMF + EVR + steroid
MMF + EVR
Tacrolimus + MMF + EVR + steroid
Tacrolimus + MMF + EVR
MMF + EVR
Cyclosporine + MMF + EVR
Cyclosporine + EVR
Cyclosporine + EVR + steroid
EVR + steroid
Tacrolimus + EVR + steroid
Tacrolimus + EVR

## Slide 4
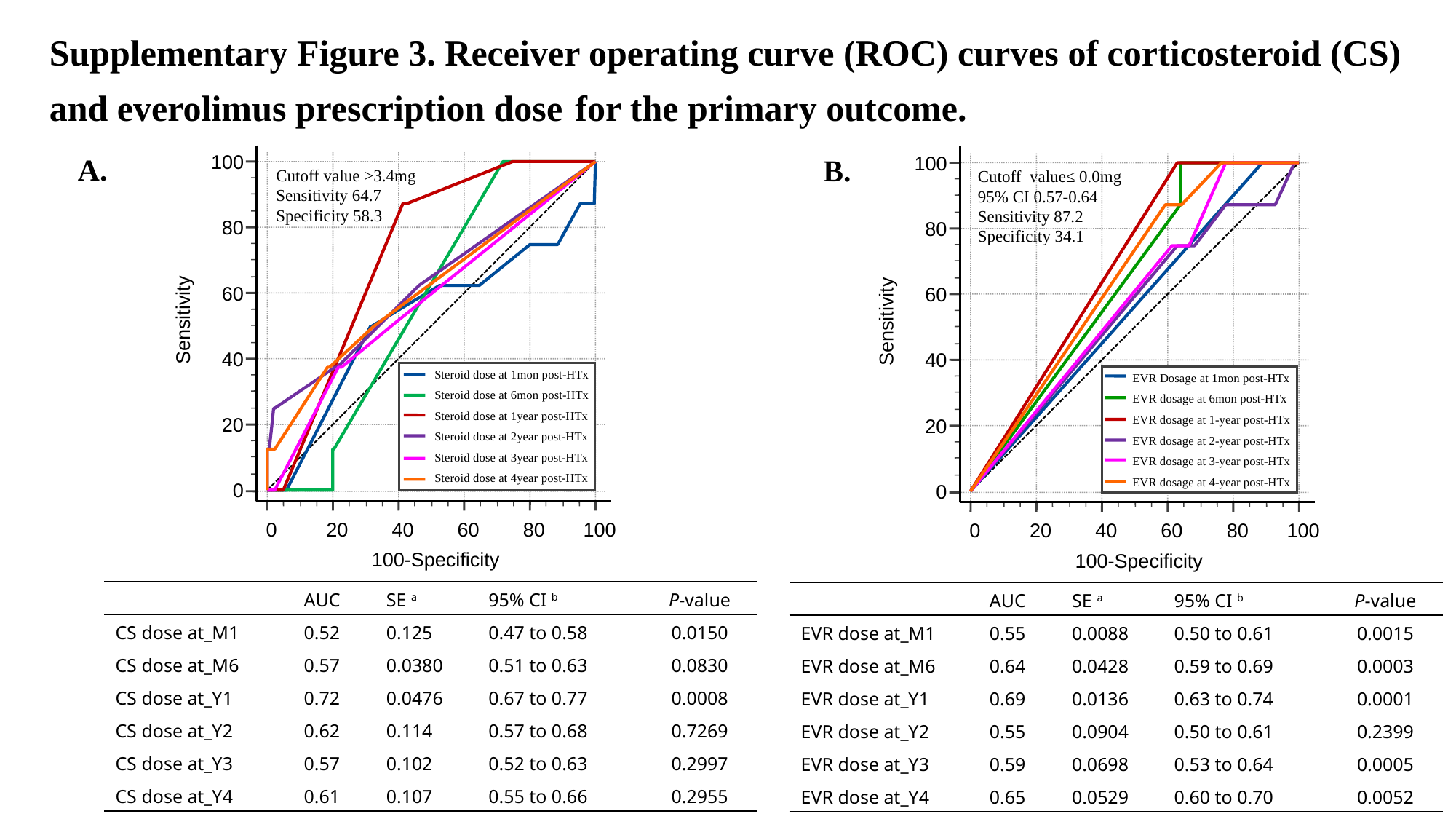

Supplementary Figure 3. Receiver operating curve (ROC) curves of corticosteroid (CS) and everolimus prescription dose for the primary outcome.
A.
100
80
60
Sensitivity
40
Steroid dose at 1mon post-HTx
Steroid dose at 6mon post-HTx
Steroid dose at 1year post-HTx
Steroid dose at 2year post-HTx
Steroid dose at 3year post-HTx
Steroid dose at 4year post-HTx
20
0
0
20
40
60
80
100
100-Specificity
B.
100
80
60
Sensitivity
40
20
0
0
20
40
60
80
100
100-Specificity
EVR Dosage at 1mon post-HTx
EVR dosage at 6mon post-HTx
EVR dosage at 1-year post-HTx
EVR dosage at 2-year post-HTx
EVR dosage at 3-year post-HTx
EVR dosage at 4-year post-HTx
Cutoff value >3.4mg
Sensitivity 64.7
Specificity 58.3
Cutoff value≤ 0.0mg
95% CI 0.57-0.64
Sensitivity 87.2
Specificity 34.1
| | AUC | SE a | 95% CI b | P-value |
| --- | --- | --- | --- | --- |
| CS dose at\_M1 | 0.52 | 0.125 | 0.47 to 0.58 | 0.0150 |
| CS dose at\_M6 | 0.57 | 0.0380 | 0.51 to 0.63 | 0.0830 |
| CS dose at\_Y1 | 0.72 | 0.0476 | 0.67 to 0.77 | 0.0008 |
| CS dose at\_Y2 | 0.62 | 0.114 | 0.57 to 0.68 | 0.7269 |
| CS dose at\_Y3 | 0.57 | 0.102 | 0.52 to 0.63 | 0.2997 |
| CS dose at\_Y4 | 0.61 | 0.107 | 0.55 to 0.66 | 0.2955 |
| | AUC | SE a | 95% CI b | P-value |
| --- | --- | --- | --- | --- |
| EVR dose at\_M1 | 0.55 | 0.0088 | 0.50 to 0.61 | 0.0015 |
| EVR dose at\_M6 | 0.64 | 0.0428 | 0.59 to 0.69 | 0.0003 |
| EVR dose at\_Y1 | 0.69 | 0.0136 | 0.63 to 0.74 | 0.0001 |
| EVR dose at\_Y2 | 0.55 | 0.0904 | 0.50 to 0.61 | 0.2399 |
| EVR dose at\_Y3 | 0.59 | 0.0698 | 0.53 to 0.64 | 0.0005 |
| EVR dose at\_Y4 | 0.65 | 0.0529 | 0.60 to 0.70 | 0.0052 |

## Slide 5
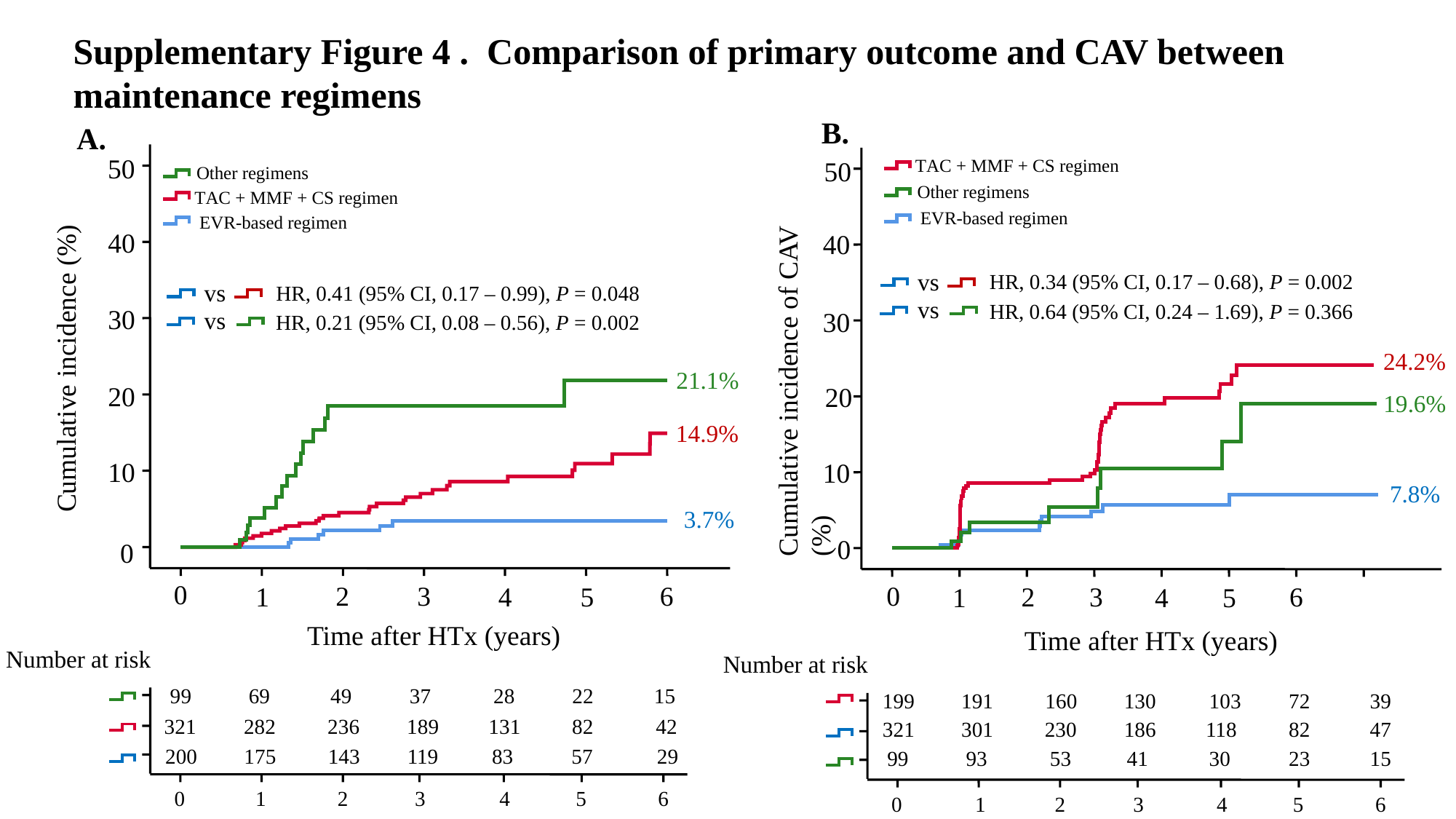

Supplementary Figure 4 . Comparison of primary outcome and CAV between maintenance regimens
B.
A.
50
TAC + MMF + CS regimen
Other regimens
EVR-based regimen
50
Other regimens
TAC + MMF + CS regimen
EVR-based regimen
40
40
HR, 0.34 (95% CI, 0.17 – 0.68), P = 0.002
vs
HR, 0.64 (95% CI, 0.24 – 1.69), P = 0.366
vs
HR, 0.41 (95% CI, 0.17 – 0.99), P = 0.048
vs
HR, 0.21 (95% CI, 0.08 – 0.56), P = 0.002
vs
30
30
Cumulative incidence (%)
Cumulative incidence of CAV (%)
24.2%
21.1%
20
20
19.6%
14.9%
10
10
7.8%
3.7%
0
0
0
0
2
3
6
2
3
6
1
4
5
1
4
5
Time after HTx (years)
Time after HTx (years)
Number at risk
99
69
49
37
28
22
15
321
282
236
189
131
82
42
200
175
143
119
83
57
29
0
1
2
3
4
5
6
Number at risk
199
191
160
130
103
72
39
321
301
230
186
118
82
47
99
93
53
41
30
23
15
0
1
2
3
4
5
6

## Slide 6
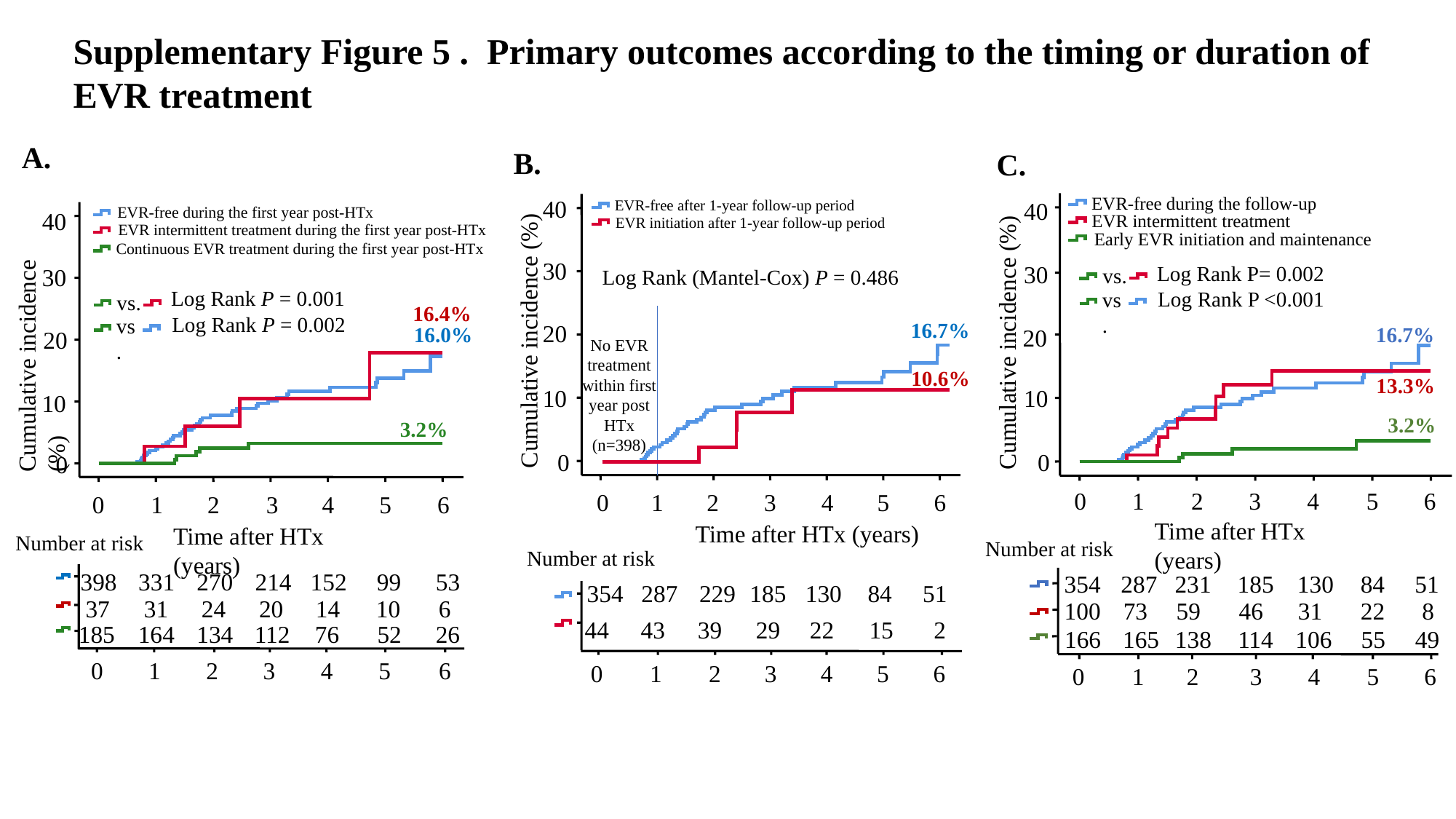

Supplementary Figure 5 . Primary outcomes according to the timing or duration of EVR treatment
A.
B.
40
EVR-free after 1-year follow-up period
 EVR initiation after 1-year follow-up period
30
Log Rank (Mantel-Cox) P = 0.486
16.7%
20
Cumulative incidence (%)
No EVR treatment within first year post HTx
(n=398)
10.6%
10
0
0
1
2
3
4
5
6
Time after HTx (years)
Number at risk
354
 287
 229
 185
 130
 84
 51
44
 43
 39
 29
 22
15
2
0
1
2
3
4
5
6
C.
EVR-free during the follow-up
40
EVR intermittent treatment
Early EVR initiation and maintenance
30
16.7%
Cumulative incidence (%)
20
13.3%
10
3.2%
0
0
1
2
3
4
5
6
Time after HTx (years)
Number at risk
354
287
231
185
130
84
51
100
73
59
46
31
22
8
166
165
138
114
106
55
49
0
1
2
3
4
5
6
EVR-free during the first year post-HTx
40
EVR intermittent treatment during the first year post-HTx
Continuous EVR treatment during the first year post-HTx
Log Rank P= 0.002
vs.
Log Rank P <0.001
vs.
30
Log Rank P = 0.001
vs.
Log Rank P = 0.002
vs.
16.4%
16.0%
20
Cumulative incidence (%)
10
3.2%
0
0
1
2
3
4
5
6
Time after HTx (years)
Number at risk
398
331
270
214
152
99
53
37
31
24
20
14
10
6
185
164
134
112
76
52
26
0
1
2
3
4
5
6

## Slide 7
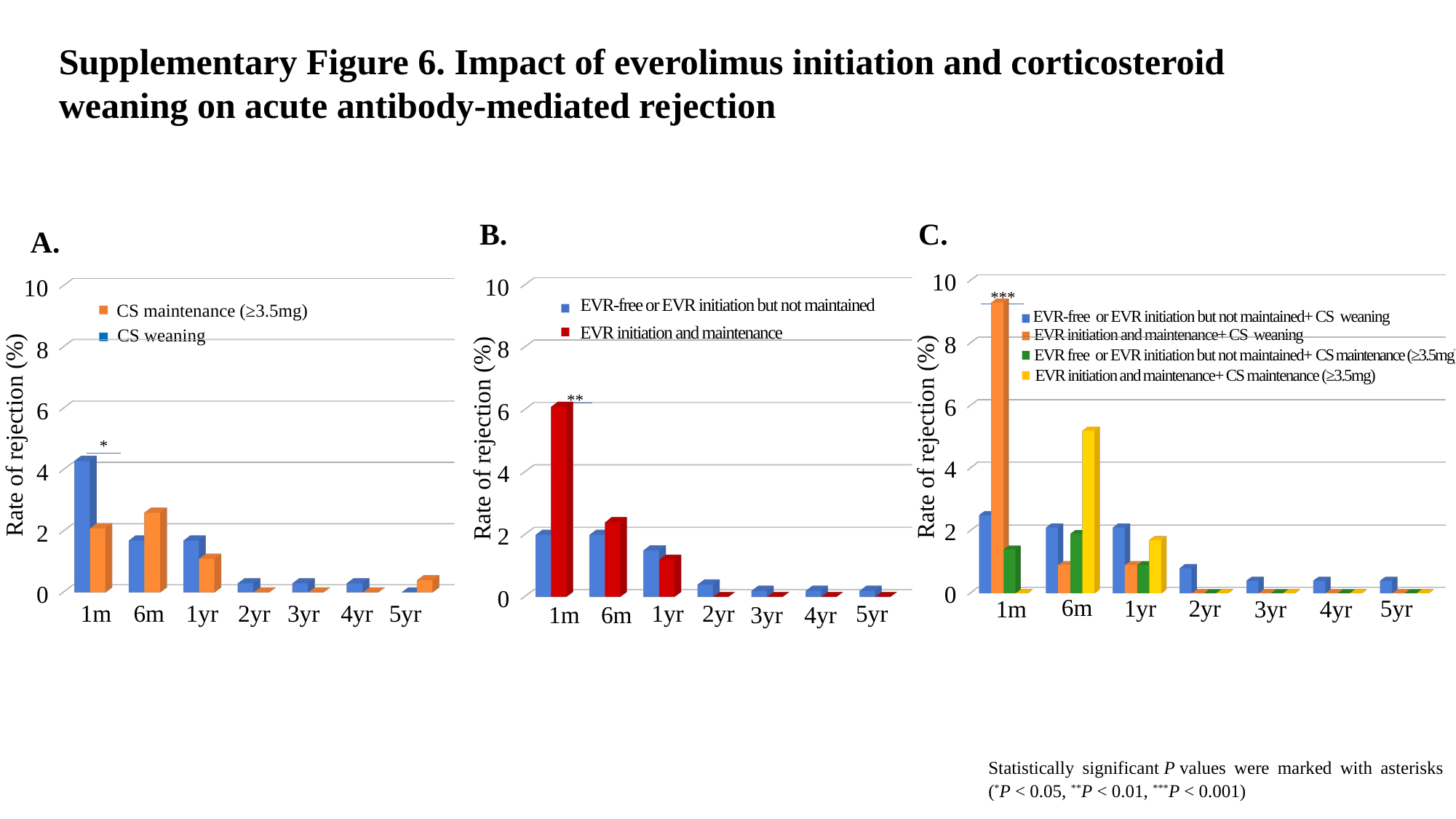

Supplementary Figure 6. Impact of everolimus initiation and corticosteroid weaning on acute antibody-mediated rejection
B.
C.
A.
[unsupported chart]
[unsupported chart]
[unsupported chart]
***
EVR-free or EVR initiation but not maintained
EVR initiation and maintenance
CS maintenance (≥3.5mg)
CS weaning
EVR-free or EVR initiation but not maintained+ CS weaning
EVR initiation and maintenance+ CS weaning
EVR free or EVR initiation but not maintained+ CS maintenance (≥3.5mg)
EVR initiation and maintenance+ CS maintenance (≥3.5mg)
**
Rate of rejection (%)
Rate of rejection (%)
Rate of rejection (%)
*
6m
1yr
2yr
5yr
1m
3yr
4yr
1m
6m
1yr
2yr
3yr
4yr
5yr
1yr
2yr
5yr
1m
6m
3yr
4yr
Statistically significant P values were marked with asterisks (*P < 0.05, **P < 0.01, ***P < 0.001)

## Slide 8
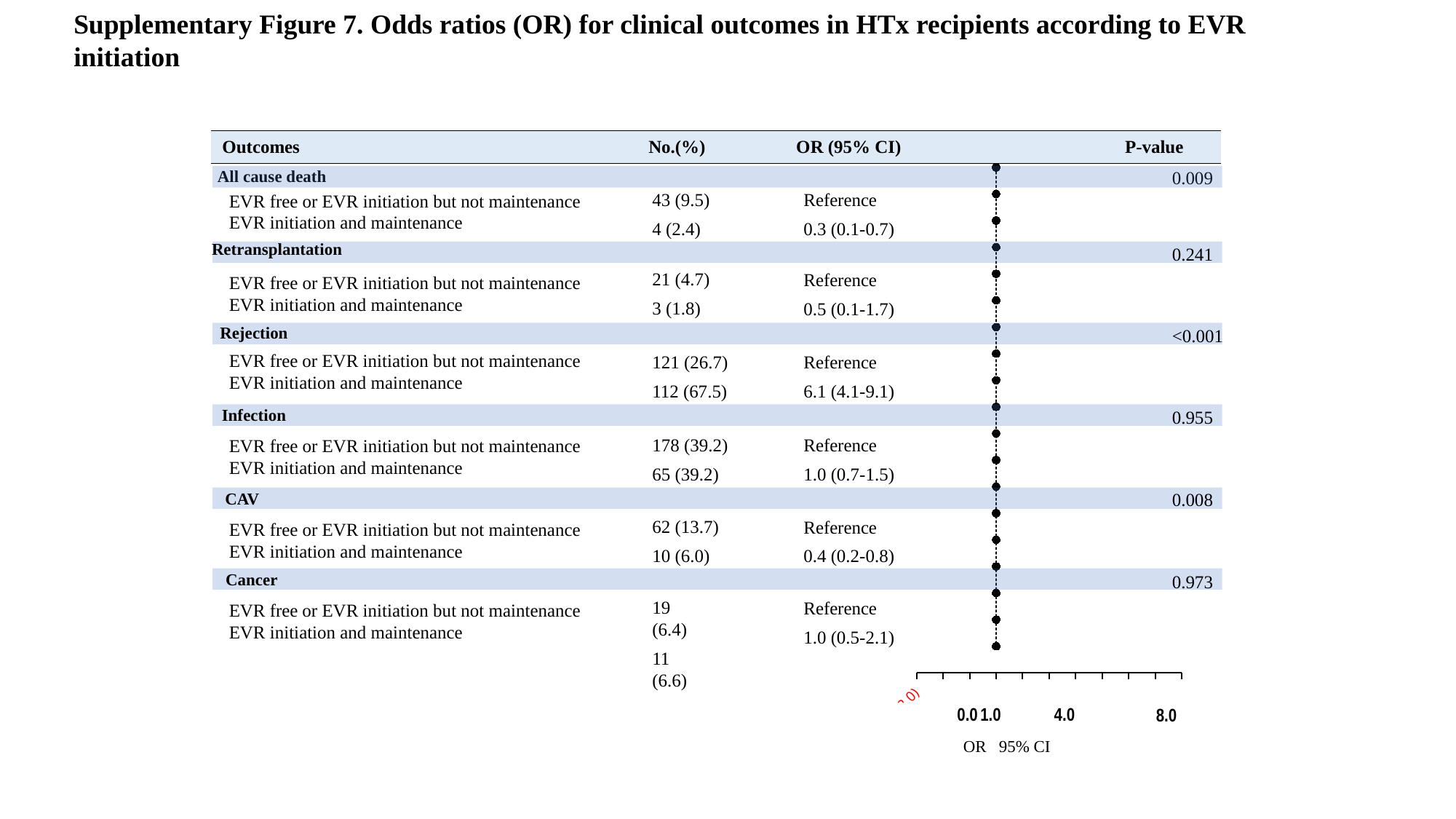

Supplementary Figure 7. Odds ratios (OR) for clinical outcomes in HTx recipients according to EVR initiation
### Chart
| Category | 19 | | |
|---|---|---|---|| Outcomes | No.(%) OR (95% CI) | | P-value |
| --- | --- | --- | --- |
| | | | |
All cause death
0.009
43 (9.5)
4 (2.4)
Reference
0.3 (0.1-0.7)
EVR free or EVR initiation but not maintenance EVR initiation and maintenance
Retransplantation
0.241
21 (4.7)
3 (1.8)
Reference
0.5 (0.1-1.7)
EVR free or EVR initiation but not maintenance EVR initiation and maintenance
Rejection
<0.001
EVR free or EVR initiation but not maintenance EVR initiation and maintenance
121 (26.7)
112 (67.5)
Reference
6.1 (4.1-9.1)
Infection
0.955
178 (39.2)
65 (39.2)
Reference
1.0 (0.7-1.5)
EVR free or EVR initiation but not maintenance EVR initiation and maintenance
0.008
CAV
62 (13.7)
10 (6.0)
Reference
0.4 (0.2-0.8)
EVR free or EVR initiation but not maintenance EVR initiation and maintenance
Cancer
0.973
19 (6.4)
11 (6.6)
Reference
1.0 (0.5-2.1)
EVR free or EVR initiation but not maintenance EVR initiation and maintenance
4.0
1.0
8.0
0.0
OR 95% CI

## Slide 9
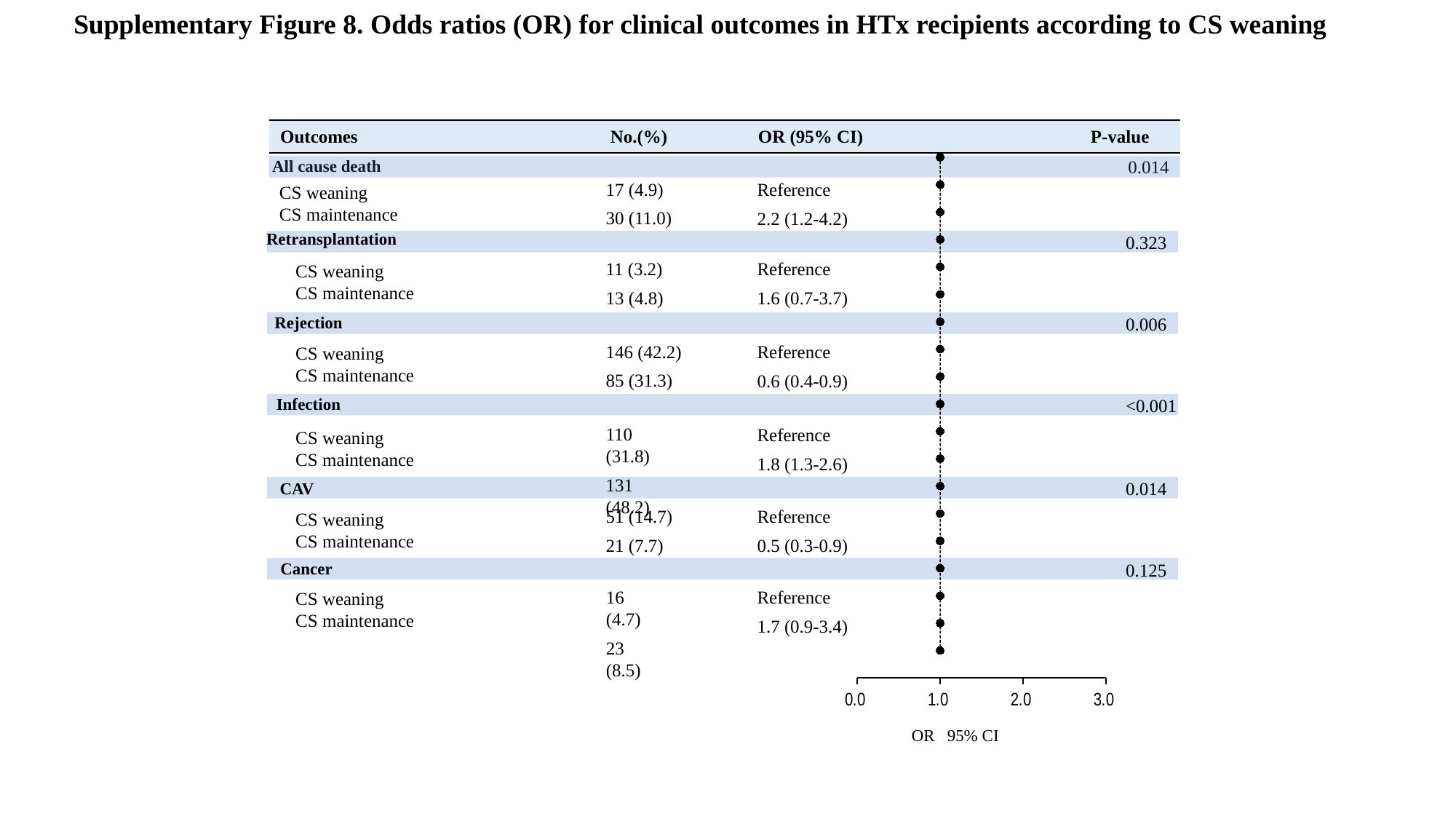

Supplementary Figure 8. Odds ratios (OR) for clinical outcomes in HTx recipients according to CS weaning
### Chart
| Category | 19 | | | | | |
|---|---|---|---|---|---|---|| Outcomes | No.(%) OR (95% CI) | | P-value |
| --- | --- | --- | --- |
| | | | |
All cause death
0.014
17 (4.9)
30 (11.0)
Reference
2.2 (1.2-4.2)
CS weaning
CS maintenance
Retransplantation
0.323
11 (3.2)
13 (4.8)
Reference
1.6 (0.7-3.7)
CS weaning
CS maintenance
Rejection
0.006
146 (42.2)
85 (31.3)
Reference
0.6 (0.4-0.9)
CS weaning
CS maintenance
Infection
<0.001
110 (31.8)
131 (48.2)
Reference
1.8 (1.3-2.6)
CS weaning
CS maintenance
0.014
CAV
51 (14.7)
21 (7.7)
Reference
0.5 (0.3-0.9)
CS weaning
CS maintenance
Cancer
0.125
16 (4.7)
23 (8.5)
Reference
1.7 (0.9-3.4)
CS weaning
CS maintenance
OR 95% CI

## Slide 10
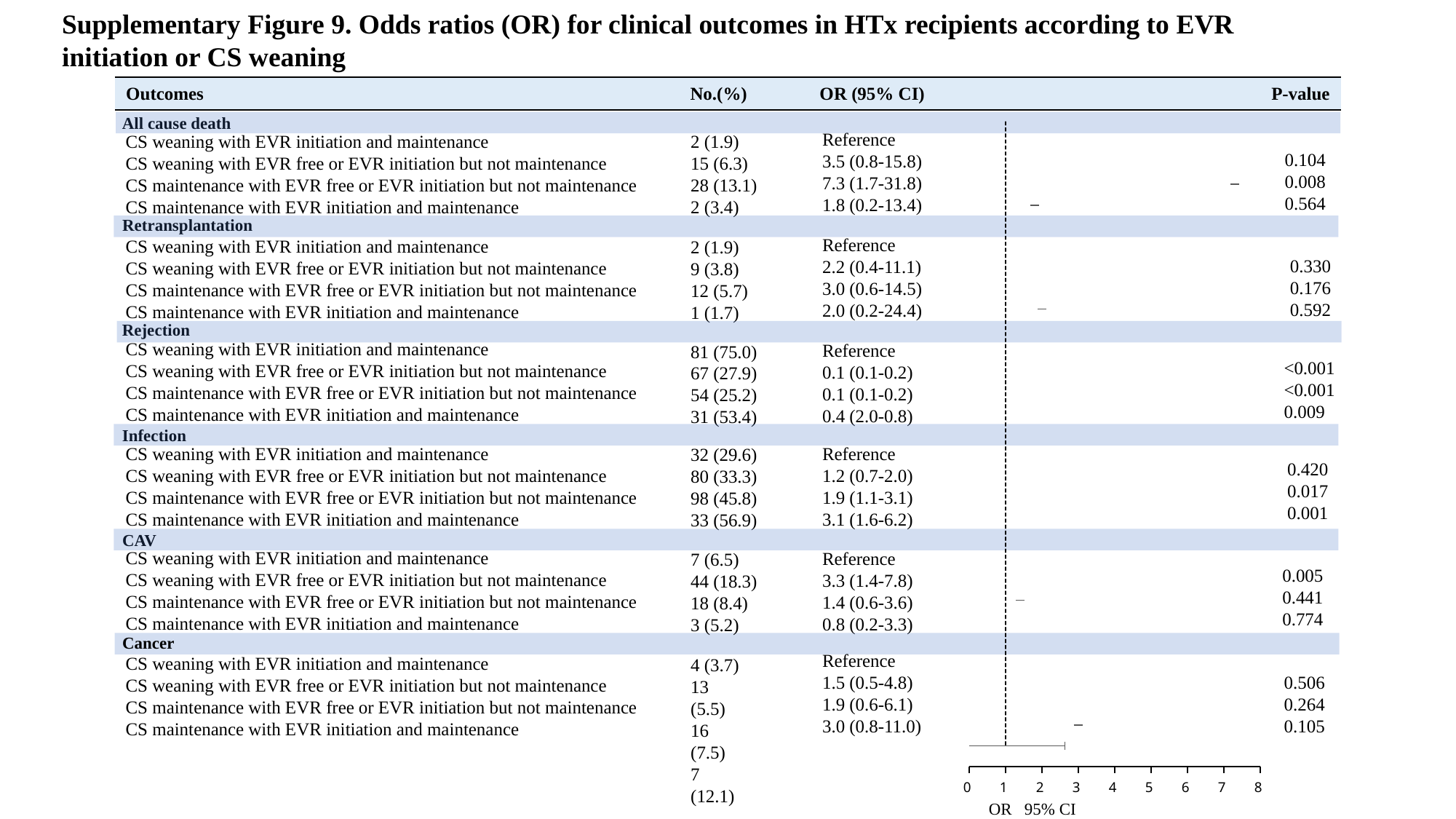

Supplementary Figure 9. Odds ratios (OR) for clinical outcomes in HTx recipients according to EVR initiation or CS weaning
### Chart
| Category | 31 | | | | | | | | | | | | | | | | |
|---|---|---|---|---|---|---|---|---|---|---|---|---|---|---|---|---|---|| Outcomes | No.(%) OR (95% CI) | | P-value |
| --- | --- | --- | --- |
| | | | |
All cause death
Reference
3.5 (0.8-15.8)
7.3 (1.7-31.8)
1.8 (0.2-13.4)
CS weaning with EVR initiation and maintenance
CS weaning with EVR free or EVR initiation but not maintenance
CS maintenance with EVR free or EVR initiation but not maintenance
CS maintenance with EVR initiation and maintenance
2 (1.9)
15 (6.3)
28 (13.1)
2 (3.4)
0.104
0.008
0.564
Retransplantation
Reference
2.2 (0.4-11.1)
3.0 (0.6-14.5)
2.0 (0.2-24.4)
CS weaning with EVR initiation and maintenance
CS weaning with EVR free or EVR initiation but not maintenance
CS maintenance with EVR free or EVR initiation but not maintenance
CS maintenance with EVR initiation and maintenance
2 (1.9)
9 (3.8)
12 (5.7)
1 (1.7)
0.330
0.176
0.592
Rejection
CS weaning with EVR initiation and maintenance
CS weaning with EVR free or EVR initiation but not maintenance
CS maintenance with EVR free or EVR initiation but not maintenance
CS maintenance with EVR initiation and maintenance
Reference
0.1 (0.1-0.2)
0.1 (0.1-0.2)
0.4 (2.0-0.8)
81 (75.0)
67 (27.9)
54 (25.2)
31 (53.4)
<0.001
<0.001
0.009
Infection
CS weaning with EVR initiation and maintenance
CS weaning with EVR free or EVR initiation but not maintenance
CS maintenance with EVR free or EVR initiation but not maintenance
CS maintenance with EVR initiation and maintenance
Reference
1.2 (0.7-2.0)
1.9 (1.1-3.1)
3.1 (1.6-6.2)
32 (29.6)
80 (33.3)
98 (45.8)
33 (56.9)
0.420
0.017
0.001
CAV
CS weaning with EVR initiation and maintenance
CS weaning with EVR free or EVR initiation but not maintenance
CS maintenance with EVR free or EVR initiation but not maintenance
CS maintenance with EVR initiation and maintenance
Reference
3.3 (1.4-7.8)
1.4 (0.6-3.6)
0.8 (0.2-3.3)
7 (6.5)
44 (18.3)
18 (8.4)
3 (5.2)
0.005
0.441
0.774
Cancer
Reference
1.5 (0.5-4.8)
1.9 (0.6-6.1)
3.0 (0.8-11.0)
CS weaning with EVR initiation and maintenance
CS weaning with EVR free or EVR initiation but not maintenance
CS maintenance with EVR free or EVR initiation but not maintenance
CS maintenance with EVR initiation and maintenance
4 (3.7)
13 (5.5)
16 (7.5)
7 (12.1)
0.506
0.264
0.105
OR 95% CI
