# Supplementary material for: Impact of Everolimus Initiation and Corticosteroid Weaning During Acute Phase After Heart Transplantation on Clinical Outcome: Data from the Korean Organ Transplant Registry (KOTRY)
Source: Transpl Int. 2024 Apr 5;37:11878. doi: 10.3389/ti.2024.11878 (PMC11028401; doi:10.3389/ti.2024.11878)
Supplement: Supplementary file 2 [file DataSheet1.docx]

**Supplemental Material**

**Impact of Everolimus Initiation and Corticosteroid Weaning During Acute Phase After Heart Transplantation on Clinical Outcome: Data From the Korean Organ Transplant Registry (KOTRY)**

**KS Lee et al.**

**Contents**

#1.Supplementary Table 1. CAV event rates and severity according to immunosuppressive regimen

#2.Supplementary Table 2. Incidence of  malignancy according to immunosuppressive regimens

#3.Supplementary Table 3. Prescription changes in immunosuppressive agents according to the presence or absence of EVR

#4.Supplementary Table 4. Difference of TAC trough levels between regimen with EVR and without EVR according to serum creatinine quartiles.

#5.Supplementary Table 5. Difference of serum creatinine levels between regimen with EVR and without EVR.

#6. The capsule sentence summary

**Supplementary Table 1. CAV event rates and severity according to immunosuppressive regimen**

| **Years post-HTx/**  **Severity of CAV** | **Total no. (%)** | **EVR-free or EVR intermittent treatment with**  **CS weaning regimen**  **(N= 240)** | **Early EVR initiation**  **and**  **maintenance with**  **CS weaning regimen**  **(N = 108)** | **EVR-free or EVR intermittent treatment with**  **CS maintenance regimen (N = 214)** | **EVR-free or EVR intermittent treatment with**  **CS maintenance regimen**  **(N = 58)** | **P-value** |
| --- | --- | --- | --- | --- | --- | --- |
| At 1-year | 34/620 (5.5) | 25/240 (10.4) | 2/108 (1.9) | 5/214 (2.3) | 2/58 (3.4) | <0.001 |
| Mild (CAV 1) | 26 | 19 | 1 | 5 | 1 |  |
| Moderate (CAV 2) | 7 | 5 | 1 | 0 | 1 |  |
| Severe (CAV 3) | 1 | 1 | 0 | 0 | 0 |  |
| At 2-years | 32/522 (6.1) | 22/176 (12.5) | 2/108 (1.9) | 6/180 (3.3) | 2/58 (3.4) | <0.001 |
| Mild (CAV 1) | 25 | 18 | 1 | 5 | 1 |  |
| Moderate (CAV 2) | 6 | 4 | 1 | 0 | 1 |  |
| Severe (CAV 3) | 1 | 0 | 0 | 1 | 0 |  |
| At 3-years | 51/414 (12.3) | 29/141 (20.6) | 6/87 (6.9) | 15/136 (11.0) | 1/50 (2.0) | 0.001 |
| Mild (CAV 1) | 38 | 22 | 3 | 13 | 0 |  |
| Moderate (CAV 2) | 12 | 7 | 3 | 1 | 1 |  |
| Severe (CAV 3) | 1 | 0 | 0 | 1 | 0 |  |
| At 4-years | 45/328 (13.7) | 28/118 (23.7) | 5/68 (7.4) | 11/100 (11.0) | 1/42 (2.4) | 0.001 |
| Mild (CAV 1) | 35 | 21 | 3 | 10 | 1 |  |
| Moderate (CAV 2) | 9 | 6 | 2 | 1 | 0 |  |
| Severe (CAV 3) | 1 | 1 | 0 | 0 | 0 |  |
| At 5-years | 35/228 (15.4) | 23/84 (27.4) | 3/41 (7.3 ) | 7/67 (10.4) | 2/36 (5.6) | 0.002 |
| Mild (CAV 1) | 29 | 19 | 2 | 7 | 1 |  |
| Moderate (CAV 2) | 4 | 3 | 0 | 0 | 1 |  |
| Severe (CAV 3) | 2 | 1 | 1 | 0 | 0 |  |
| At 6-years | 21/143 (14.7) | 13/53 (24.5) | 4/23 (17.4) | 4/42 (9.5) | 0/25 (0.0%) | 0.024 |
| Mild (CAV 1) | 19 | 12 | 3 | 4 | 0 |  |
| Moderate (CAV 2) | 1 | 1 | 0 | 0 | 0 |  |
| Severe (CAV 3) | 1 | 0 | 1 | 0 | 0 |  |

Abbreviations: CAV, cardiac allograft vasculopathy; CS, corticosteroid; HTx, heart transplantation; EVR: everolimus

**Supplementary Table 2. Incidence of  malignancy according to immunosuppressive regimens**

| **Outcomes** | **Total**  **Events- no. (%)** | **EVR-free or**  **EVR intermittent**  **treatment with CS weaning regimen (N= 240)** | **Early EVR initiation**  **and maintenance with**  **CS weaning regimen**  **(N = 108)** | **EVR-free or**  **EVR intermittent**  **treatment with**  **CS maintenance**  **regimen (N = 214)** | **Early EVR initiation**  **and maintenance with**  **CS maintenance regimen (N = 58)** | ***P* value** |
| --- | --- | --- | --- | --- | --- | --- |
| Malignancy (months post-HTx) |  |  |  |  |  |  |
| 1 | 9/620 (1.5%) | 3/240 (1.3%) | 0/108 (0.0%) | 5/214 (2.3%) | 1/58 (1.7%) | 0.414 |
| 6 | 10/620 (1.6%) | 4/240 (1.7%) | 0/108 (0.0%) | 5/214 (2.3%) | 1/58 (1.7%) | 0.478 |
| 12 | 10/620 (1.6%) | 4/240 (1.7%) | 0/108 (0.0%) | 6/214 (2.8%) | 0/58 (0.0%) | 0.026 |
| 24 | 12/511 (2.3%) | 3/173 (1.7%) | 0/108 (0.0%) | 8/172 (4.7%) | 1/58 (1.7%) | 0.179 |
| 36 | 15/411 (3.6%) | 5/139 (3.6%) | 2/87 (2.3%) | 6/135 (4.4%) | 2/50 (4.0%) | 0.849 |
| 48 | 15/326 (4.6%) | 5/117 (4.3%) | 2/68 (2.9%) | 6/99 (6.1%) | 2/42 (4.8%) | 0.816 |
| 60 | 9/227 (4.0%) | 5/84 (6.0%) | 0/41 (0.0%) | 3/66 (4.5%) | 1/36 (2.8%) | 0.431 |

Abbreviations: CS, corticosteroid; HTx, heart transplantation; EVR: everolimus

**Supplementary Table 3. Prescription changes in immunosuppressive agents according to the presence or absence of EVR**

| **At discharge after HTx** | **EVR initiation and maintenance regimen** | **EVR-free or EVR intermittent treatment regimen** | ***P* value** |
| --- | --- | --- | --- |
| TAC–no. (%) | 63 (86.3) | 676 (94.5) | 0.011 |
| CsA–no. (%) | 7 (9.6) | 20 (2.8) | 0.009 |
| MMF–no. (%) | 40 (54.8) | 638 (89.2) | <0.001 |
| CS–no. (%) | 65 (89.0) | 676 (94.5) | 0.068 |
| **At 1 month after HTx** | **EVR initiation and maintenance regimen** | **EVR-free or EVR intermittent treatment regimen** | ***P* value** |
| TAC–no. (%) | 54 (88.5) | 681 (96.7) | 0.007 |
| CsA–no. (%) | 7 (11.5) | 17 (2.4) | 0.002 |
| MMF–no. (%) | 34 (55.7) | 624 (88.6) | <0.001 |
| CS–no. (%) | 61 (100) | 691 (98.2) | 0.614 |
| **At 6 months after HTx** | **EVR initiation and maintenance regimen** | **EVR-free or EVR intermittent treatment regimen** | ***P* value** |
| TAC–no. (%) | 193 (92.4) | 450 (96.4) | 0.051 |
| CsA–no. (%) | 10 (4.8) | 16 (3.4) | 0.392 |
| MMF–no. (%) | 168 (80.8) | 435 (93.1) | <0.001 |
| CS–no. (%) | 97 (46.6) | 420 (89.9) | <0.001 |
| **At 12 months after HTx** | **EVR initiation and maintenance regimen** | **EVR-free or EVR intermittent treatment regimen** | ***P* value** |
| TAC–no. (%) | 185 (92.0) | 400 (95.5) | 0.095 |
| CsA–no. (%) | 10 (5.0) | 18 (4.3) | 0.684 |
| MMF–no. (%) | 163 (81.1) | 397 (94.7) | <0.001 |
| CS–no. (%) | 83 (41.3) | 354 (84.5) | <0.001 |
| **At 24 months after HTx** | **EVR initiation and maintenance regimen** | **EVR-free or EVR intermittent treatment regimen** | ***P* value** |
| TAC–no. (%) | 170 (91.4) | 312 (96.0) | 0.045 |
| CsA–no. (%) | 10 (5.4) | 13 (4.0) | 0.509 |
| MMF–no. (%) | 130 (69.9) | 309 (95.1) | <0.001 |
| CS–no. (%) | 64 (34.4) | 173 (53.2) | <0.001 |
| **At 36 months after HTx** | **EVR initiation and maintenance regimen** | **EVR-free or EVR intermittent treatment regimen** | ***P* value** |
| TAC–no. (%) | 140 (92.1) | 250 (96.5) | 0.063 |
| CsA–no. (%) | 10 (6.6) | 9 (3.5) | 0.153 |
| MMF–no. (%) | 103 (67.8) | 248 (95.8) | <0.001 |
| CS–no. (%) | 48 (31.6) | 114 (44.0) | 0.016 |
| **At 48 months after HTx** | **EVR initiation and maintenance regimen** | **EVR-free or EVR intermittent treatment regimen** | ***P* value** |
| TAC–no. (%) | 113 (88.3) | 191 (96.5) | 0.006 |
| CsA–no. (%) | 10 (7.8) | 7 (3.5) | 0.124 |
| MMF–no. (%) | 78 (60.9) | 185 (93.4) | <0.001 |
| CS–no. (%) | 38 (29.7) | 68 (34.3) | 0.399 |

Abbreviations:  HTx, heart transplantation; CS, corticosteroid; CsA, cyclosporine; EVR, everolimus; MMF, mycophenolic mofetil; TAC, tacrolimus

**Supplementary Table 4. Difference of TAC trough levels between regimen with EVR and without EVR according to serum creatinine quartiles.**

|  | **Early EVR initiation and maintenance regimen** | | | **EVR free or EVR intermittent treatment regimen** | | |
| --- | --- | --- | --- | --- | --- | --- |
|  | TAC trough level (ng/mL) | |  | TAC trough level (ng/mL) | |  |
| Months post-HTx | Serum Cr Q1-Q2 | Serum Cr Q3-Q4 | P-vale | Serum Cr Q1-Q2 | Serum Cr Q3-Q4 | P-value |
| At 1month | 7.5±2.3 | 7.5±3.5 | 0.913 | 8.9±3.3 | 8.9±3.0 | 0.891 |
| At 6months | 5.2±2.2 | 5.0±2.5 | 0.425 | 8.9±3.3 | 8.2±2.8 | 0.014 |
| At 12months | 4.8±1.6 | 4.5±1.9 | 0.211 | 8.1±3.0 | 7.8±3.2 | 0.331 |
| At 24months | 4.6±1.5 | 4.9±2.0 | 0.198 | 7.3±2.6 | 6.9±2.4 | 0.166 |
| At 36months | 4.2±2.5 | 4.0±2.5 | 0.515 | 6.7±2.3 | 6.1±1.8 | 0.030 |
| At 48months | 4.4±1.5 | 4.3±2.0 | 0.810 | 6.7±2.0 | 6.0±1.9 | 0.022 |
| At 60months | 4.0±1.7 | 3.8±1.2 | 0.511 | 6.6±1.9 | 5.7±1.7 | 0.006 |

Abbreviations: HTx, heart transplantation; TAC, tacrolimus; EVR, everolimus; Cr, creatinine; Q1, quartile 1; Q2, quartile 2; Q3, quartile 3; Q4, quartile 4

**Supplementary Table 5. Difference of serum creatinine levels between regimen with EVR and without EVR.**

| **Renal function**  Serum creatinine (mg/dL) | **Early EVR initiation and maintenance regimen** | **EVR free or EVR intermittent treatment regimen** | **P-value** |
| --- | --- | --- | --- |
| At discharge | 1.30±1.03 | 1.25±0.97 | 0.529 |
| At 1month | 0.96±0.55 | 1.18±0.88 | <0.001 |
| At 6months | 1.19±0.84 | 1.40±0.79 | 0.002 |
| At 12months | 1.17±0.75 | 1.37±0.79 | 0.003 |
| At 24months | 1.16±0.70 | 1.35±0.98 | 0.019 |
| At 36months | 1.20±0.90 | 1.31±0.83 | 0.196 |
| At 48months | 1.08±0.37 | 1.21±0.60 | 0.025 |
| At 60months | 1.15±0.43 | 1.26±0.54 | 0.123 |

Abbreviations: EVR, everolimus

**Capsule Sentence Summary**

The early EVR initiation and CS weaning within the first year post-HTx is associated with reduced the risk of primary adverse events and cardiac allograft vasculopathy (CAV). However, this regimen may increase the risk of acute allograft rejection during the acute phase post-HTx.
